# Neuronal correlates of spider phobia in a combined fNIRS-EEG study

**DOI:** 10.1038/s41598-020-69127-3

**Published:** 2020-07-28

**Authors:** David Rosenbaum, Elisabeth J. Leehr, Agnes Kroczek, Julian A. Rubel, Isabell Int-Veen, Kira Deutsch, Moritz J. Maier, Justin Hudak, Andreas J. Fallgatter, Ann-Christine Ehlis

**Affiliations:** 1 Department of Psychiatry and Psychotherapy, University Hospital of Tuebingen, Calwerstraße 14, 72076 Tübingen, Germany; 2 Department of Psychiatry, University of Muenster, Muenster, Germany; 3 Department of Psychotherapy Research, Justus-Liebig-University Giessen, Giessen, Germany; 4 Center on Mindfulness and Integrative Health Intervention Development, University of Utah, Salt Lake City, UT 84112 USA; 5 LEAD Graduate School and Research Network, University of Tuebingen, Tuebingen, Germany

**Keywords:** Psychology, Human behaviour, Psychiatric disorders, Cognitive neuroscience, Emotion

## Abstract

Specific phobia is associated with aberrant brain activation in confrontation paradigms with phobic stimuli. In previous EEG research enhanced event-related potentials (ERPs) in the late-positive potential (LPP) window have been observed. Further, studies with functional near-infrared spectroscopy (fNIRS) and fMRI suggest that spider phobia is associated with enhanced activation within cortical and subcortical areas. In the current study we investigated the neuronal correlates of spider phobia in a combined fNIRS–EEG study. To this end, 37 spider phobic patients (PP) and 32 healthy controls (HC) underwent a symptom provocation paradigm during which subjects watched video clips of spiders and domestic animals (confrontation phase) after being primed on the content of the video (anticipation phase). Simultaneously, fNIRS, EEG, electromyography (EMG), electrocardiography and behavioral measures were assessed. Results showed increased LPP amplitudes, increased hemodynamic responses in the cognitive control network, and increased EMG activity and heart rate during spider conditions in PP in comparison to HC. Furthermore, in behavioral ratings PP showed higher emotional distress and avoidance. Behavioral ratings, fNIRS and EEG data showed positive correlations on a between-subject as well as on a within-subject level. Our results merge the existing data on neurophysiological correlates of phobic stimulus processing in hemodynamic and electrophysiological research and extend those of static visual material (pictures) to dynamic visual material (videos).

## Introduction

Spider phobia is one of the most prevalent specific phobias with especially high point prevalence in women^1^. While many studies examine the effects of different treatments, it is necessary to investigate the neuronal correlates of spider phobia to come to terms with underlying biological mechanisms which then may be used in clinical application. In 18.7% of those individuals with any specific phobia, it leads to a significant impairment in functioning^1^. Although effective first-line treatments have been established, e.g. exposure-based cognitive behavioral therapy, about one third of patients do not respond with significant fear reduction or experience a return of fear after short-term relief^2^. Investigating the neurobiology of fear stimuli processing might allow the identification of aberrations on a neural level which could help to tailor psychotherapeutic interventions^3^.

The study of physiological fear response has a long history because of the easy operationalization of fear-induction. Fear is accompanied by a strong physiological response that has been discussed at different processing stages within the threat imminence model^4^. Importantly, the fear response might already start with the anticipation of fear cues and differ during different process stages^5^. Electroencephalography (EEG) is a convenient method for measuring the neurophysiological underpinnings of cognitive processes related to the fear response, with several studies providing consistent evidence for an enhanced P300 amplitude and late positive potential (LPP) for adult arachnophobic patients, but not for matched healthy controls^6–9^ when presented with phobic stimuli. The increased amplitude of the P300 and LPP is often interpreted in terms of increased selective attention. Furthermore, phobic fear has been differentiated from disgust and general fear. Schienle et al.^10^ were able to show that P300 amplitudes decreased from phobic stimuli (spiders) to disgust and fear stimuli to neutral stimuli, whereas all comparisons were significant except for disgust and fear. Additionally, neutral pictures, in contrast to pictures associated with disgust, fear and phobic pictures, elicited smaller LPP amplitudes. Interestingly, the effect of increased P300 amplitudes significantly increased from frontal to central to parietal sites whereas the effect of higher LPP amplitudes significantly increased from parietal to central to frontal cortices. These results have been found to be not specifically associated with spider phobia or phobia in general but with attention allocation and, more important in this context, emotional significance^6^. This is in accordance with theories suggesting an impact of phobia on different levels of consciousness, meaning that phobic stimuli are differently encoded, interpreted and decoded. Furthermore, emotional stroop paradigms show enhanced P300 amplitudes when phobic subjects are confronted with phobic stimuli in contrast to neutral ones^11^. Interestingly, with respect to treatment effects and effects of repeated exposure to phobic stimuli, contradictory LPP results were observed. In a study of a single session experiment with repeated presentations of spider phobic stimuli, Michalowski et al.^7^ observed a decrease of LPP amplitudes in spider phobic subjects. In contrast, in two studies on the effects of exposure therapy on a symptom provocation paradigm in children^12^ and adults^6^, Leutgeb et al. reported increases in LPP amplitudes after treatment. The paradox of increased LPP amplitudes after successful treatment with cognitive behavioral therapy (CBT) was explained by an allocation of directed attentional resources, while the decreased LPP amplitudes of the study of Michalowski et al.^7^ might reflect emotion suppression strategies.

Further, there is a rich literature on hemodynamic responses during confrontation with fearful material from fMRI studies. In their meta-analysis, Peñate et al.^13^ investigated fMRI studies of healthy controls and phobic individuals, finding increased brain activity in the left amygdala, insular cortex, fusiform gyrus, dorsolateral prefrontal cortex and left cingulate cortex in the latter when presented with phobic stimuli. Interestingly, Ipseret al.^14^ found a similar pattern comprising higher activations in bilateral insula, amygdala, right medial and right superior frontal cortex and extrastriate visual cortex. Similar results were observed with respect to fNIRS. For example, Deppermann et al. reported increased functional connectivity between prefrontal areas in spider phobic patients (PP) during an emotional stroop task^15,16^. Additionally, Landowska et al.^17^ aimed to measure brain activity using virtual reality in participants with moderate acrophobia. They were able to show increased brain activity in the course of the experiment, primarily in the medial prefrontal cortex, when participants were confronted with a pit room (virtual heights condition) in contrast to a training room (control condition). Köchel et al.^18^ found increased premotor cortex activation in patients with dental phobia, compared to non-phobics, when being presented the sound of a dental drill. Furthermore, Tuscan et al.^19^ were able to show right-sided prefrontal activation in socially phobic patients during ecologically valid social tasks using fNIRS. In summary, confrontation with phobic material seems to elicit widespread activation in subcentral areas involved in the fear response and emotional processing, as well as cortical regions associated with cognitive control (lateral PFC), emotion regulation (medial PFC), action preparation (premotor cortex) and general stimulus processing. These regions form two brain networks relevant for the processing of phobic stimuli: The fear-network (e.g. amygdala, thalamus, hippocampus, sensory areas) relevant for fear conditioning and recognition of phobic stimuli^20,21^ and the cognitive control network (e.g. dorsolateral prefrontal cortex, inferior prefrontal gyrus and angular gyrus) relevant for cognitive control and model-based emotion regulation^22–27^.

With respect to spider phobia, combined measurements of fMRI and EEG have been analyzed in a study of Michalowski et al., in which neural response patterns in spider phobic, blood-injection injury and socially fearful individuals in response to fear-specific, as well as neutral stimuli, were investigated^8^. Fear-specific stimuli elicited larger LPP’s than neutral pictures, especially in the respective fearful individuals. Fearful individuals showed an increased P1 amplitude regardless of stimulus category, which was interpreted as hypervigilance effect. All fear groups showed increased brain responses for fear-specific stimuli compared to the control group and in most cases also with respect to the two other fear control groups.

There are several studies assessing EEG and fNIRS simultaneously to investigate whether the combination of electrophysiological and hemodynamic data enables a better understanding of brain activity and may reveal biomarkers for specific disorders. So far, findings are emerging^28,29^ as correlations are found throughout several studies. For example, Sun et al. investigated the neural correlates of automatic facial expressions when assessing EEG and fNIRS simultaneously^30^. The complimentary information provided by combining fNIRS with EEG outperforms methods using the imaging methods in isolation. fNIRS measurement allows higher spatial resolution of cortical activity involved in cognitive control of emotional processes providing additional information to clarify the reported inconsistent results. For example, Al-Shargie et al. found a significant improvement of stress detection when combining fNIRS and EEG^31^. Further, results of another study show that both signals correlate intrinsically, especially for low-frequency theta and delta bands^32^. Assessing fNIRS and EEG provides an immense advantage compared to fMRI in regard to insensitivity to movement artifacts and electrical noise^33–35^. fNIRS is a convenient method to assess processing of fearful stimuli in phobics^16,36^, despite potential fright reactions with inevitable movement- and arousal-artifacts. Further, it can be measured simultaneously to EEG, as the measurements do not interfere with each other^37^. However, multi-method studies on the subject of arachnophobia are rare and the associations between different physiological measurements so far are not perfectly understood. As fNIRS and EEG are economic and ecologically valid measurements in the study of psychopathology and the neuronal correlates of interventions, this study aimed to investigate the hemodynamic and electrophysiological correlates of arachnophobia. Understanding the processing of the feared stimulus, e.g. spiders, on a neural level might give indication for treatment optimization.

Therefore, we conducted a simultaneous fNIRS-EEG study during exposure to a phobic stimulus (spider) in spider phobics, aiming to capture the neuronal correlates of spider phobia via hemodynamic and electrophysiological changes. To capture the autonomous part of the fear response, we additionally measured heart rate via electrocardiogram (ECG) and facial muscle activation via electromyography (EMG). We operationalized the induction of fear by using priming through cues in an anticipation phase and a confrontation phase with video clips of either spiders or domestic animals (control). After each trial, subjects rated their emotional distress and avoidance during the trial. In line with the rich literature on the topic, we expected to find elevated LPP amplitudes and increased hemodynamic responses in the cognitive control network (CCN) in spider phobic patients (PP) in comparison to healthy controls (HC) during the processing of phobia-related stimuli. Furthermore, we expected that subjects would show enhanced emotional distress and avoidance on a behavioral level, as well as on a physiological level by means of heart rate and facial expression. Lastly, we hypothesized that EEG, fNIRS and behavioral markers would be positively related.

## Material and methods

### Participants

The ethics committee at the University Hospital and University of Tuebingen approved this project and all subjects gave written informed consent. All methods and procedures used in this study were in accordance with current guidelines of the World Medical Association Declaration of Helsinki. For this study, n = 37 arachnophobic patients (PP) and n = 32 healthy controls (HC) were recruited. Due to technical problems, fNIRS data was missing in 7 HC and 7 PP; EEG data was missing in 6 HC and 2 PP. Subjects were recruited via flyers and email. The study protocol is registered at ClinicalTrials.gov (NCT03653923). Part of this study has been published elsewhere^38^. Exclusion criteria were acute physical illness, neurological disorders, substance abuse, chronic or acute diseases that affect brain functioning such as diabetes or kidney failure, cardiac arrhythmia or other cardiac diseases. HC and PP did not differ in sex [*χ*^2^ = 0.477, *p* > 0.1] and years of education [*t*(58) = 1.599, *p* > 0.1]. However, both groups differed in age significantly [*t*(60) = 2.65, *p* < 0.01] (HC: *M* = 24.22, *SD* 4; PP: *M* = 28.74, *SD* 4.16). The sample was 85% female and the average duration of education was 17 years (*SD* 4.00 years).

All PP fulfilled criteria for specific phobia/arachnophobia according to the Structured Clinical Interview for DSM-IV (SCID)^39^. Comorbid diagnoses in the clinical sample were: previous episode of major depressive disorder (10.81%) and other phobic disorders (29.70%) (e.g. acrophobia, panic disorder). Three subjects had undergone psychotherapeutic treatment prior to their participation in the study. PP and HC differed significantly in spider phobia related symptoms, which were assessed with the Spider Phobic Questionnaire (SPQ), Spider Beliefs Questionnaire (SBQ) and the Fear of Spiders Questionnaire (FSQ), as well as in measures of behavioral avoidance (BAT)^40–42^ (see Table 1). BAT assessment before treatment indicated relevant fear and avoidance in the PP: 33.3% of the sample were maximally able to watch a spider in a jar from a distance of 5 m, 50% from a distance of 2 m, 6.7% from a distance of 0.5 m, 10% were able to look at the spider from the near distance (1 m) until fear and avoidance were no longer tolerable. No PP was able to touch the spider with a hand or pen. In contrast, all HC scored maximum points on the BAT, letting the spider walk up their arm.

**Table 1:** Differences in spider phobia related symptoms between the groups.

	Mean HC	Mean PP	t	df	p	d
SBQ-mean	5.6	54.0	23.019	49.306	< 0.001	5.48
SBQ-unpredictability	23.6	83.5	15.653	44.826	< 0.001	4.06
SBQ-territory	5.5	60.7	16.925	47.487	< 0.001	4.02
SBQ-hunter and prey	2.0	29.5	8.505	39.641	< 0.001	1.99
SBQ-aggression	3.5	21.4	5.857	40.328	< 0.001	1.37
SBQ-multiplication	4.1	30.2	6.636	44.319	< 0.001	1.57
SBQ-panic	1.5	65.9	27.038	39.748	< 0.001	6.33
SBQ-paralisis	0.6	41.0	7.478	36.369	< 0.001	1.74
SBQ-incubation	1.9	53.8	12.283	39.276	< 0.001	2.87
FSQ	0.2	4.1	26.261	40.842	< 0.001	6.35
SPQ	3.2	20.8	34.888	52.621	< 0.001	5.76
BAT	11.0	2.8	− 34.888	31	< 0.001	− 8.70

*SPQ* Spider Phobia Questionnaire, *SBQ* Spider Beliefs Questionnaire, *FSQ* Fear of Spiders Questionnaire, *BAT* Behavioral Avoidance Test.

### Experimental procedure

**Figure 1:**
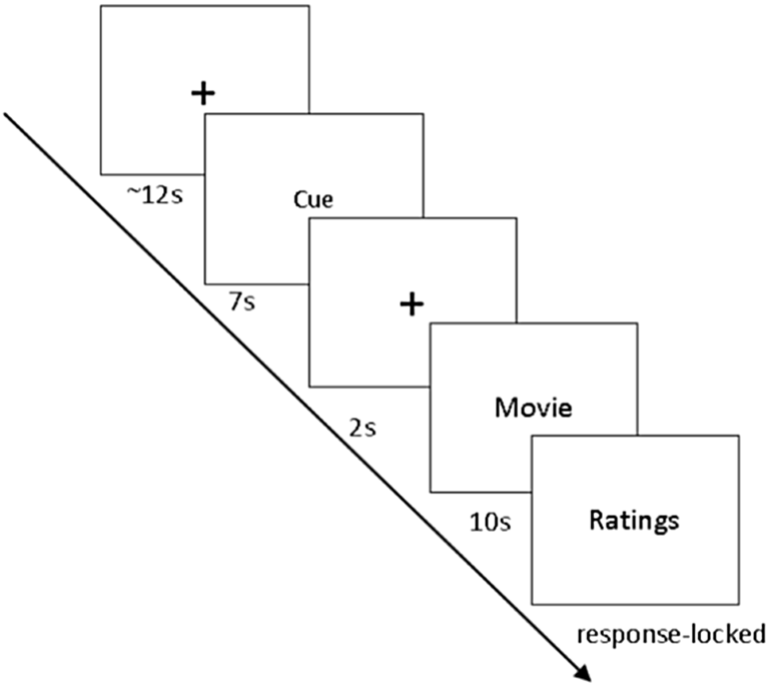
Flow chart of the experimental paradigm.

In this study, we report the comparisons of a multimodal fNIRS-EEG-EMG-ECG study in PP and HC. The data was selected from a larger study design in which fNIRS is used to track changes in situ during the treatment of spider phobia. The current paper addresses differences between HC and PP during a symptom provocation paradigm that was assessed before the treatment of the PP. Before the measurement, inclusion criteria were assessed via telephone interviews. The day of the measurement, subjects were informed about the experimental procedure and provided signed informed consent. Afterwards, the BAT was administered, followed by symptom questionnaires and demographic data collection. While subjects completed the questionnaires, the EEG-fNIRS caps, EMG and ECG were prepared for measurement. During the paradigm, subjects were instructed to watch video clips showing spiders or domestic animals for 10 s. Videos were taken from documentations about spiders and domestic animals. Spider videos showed different spiders native to Europe. Control videos showed dogs, cats and sheep. The video material is available on demand from the first author. The average rating per video is presented in the supplemental material. The participants watched 40 video clips in total in a randomized order with 50% being control trials (showing domestic animals) and 50% being experimental trials (showing spiders). Each trial consisted of a fixation cross jittered between 10 and 15 s, followed by a 7 s cue that informed the subjects about the trial that would follow (spider or domestic animal; anticipation phase). Afterwards, a fixation cross was presented for 2 s before the video trial started, which lasted for 10 s. Then, the subjects rated their emotional distress, as well as how much they avoided their feelings while watching the videos on a Likert scale ranging from 1 to 9 (see Fig. 1). Ratings were given during each trial after watching the video clip and the duration of the rating phase was response-locked. In the whole experiment, 120 different video clips were used (60 spider and 60 neutral) in 3 different versions of the paradigm. Versions were randomized between HC and PP (see supplemental material).

### fNIRS

fNIRS data was assessed as described in previous studies of our group^26,43–45^. Oxygenated (O_2_Hb) and deoxygenated (HHb) hemoglobin levels (concentration changes) were assessed with a continuous wave, multichannel fNIRS system (ETG-4000 Optical Topography System; Hitachi Medical Co., Japan) with a temporal resolution of 10 Hz. Data was recorded with a semiconductor laser and avalanche diodes at two wavelengths (695±20 and 830±20 nm) with 4.0.2 mW for each wavelength at each optode. Optodes were placed on an electrode cap with EEG and fNIRS holders (see supplementary Figure S1). We used two frontal and one parietal probeset as described in Rosenbaum et al.^46^ with reference points at F3 and F4 for the frontal probesets and Pz for the parietal probeset according to the international 10–20 system. The fNIRS placement allowed the assessment of the bilateral inferior frontal gyrus (IFG), bilateral dorsolateral prefrontal cortex (DLPFC) and the superior parietal lobule (SPL). Corresponding brain areas of each channel were extrapolated from reference points based on the Colin 27 template^47–49^).

Electrophysiological measurements were assessed with Brain Products Soft- and Hardware (BrainAmp ExG amplifier, Brain Vision Recorder) at 1,000 Hz. We used standard Ag/AgCl EEG ring electrodes of 8 mm diameter. EEG was recorded for electrodes F3, Fz, F4, C3, Cz, C4, P3, Pz, P4 online against FCz. Electrode placement was done according to the international 10–20 system^46^. Further, bilateral mastoids were used for offline re-referencing. Vertical and horizontal electrooculogram (VEOG/HEOG), electromyogram (EMG) and electrocardiogram (ECG) were additionally assessed with Brain Products Soft- and Hardware. EMG was assessed with two standard Ag/AgCl ring electrodes of 8 mm diameter placed on the corrugator supercilii over the left eye. ECG was assessed as in Rosenbaum et al.^26^. The signal of ECG and EMG was recorded using BrainAmp ExG amplifier and Brain Vision Recorder software.

### Preprocessing

After the export of the fNIRS data, data were preprocessed in MATLAB 2017a in the following way: absorbed NIR-light was transformed into O_2_Hb and HHb levels by means of a modified Beer-Lambert law, we used Temporal Derivative Distribution Repair (TDDR) for movement artefact correction of sharp peaking signals^50^, band-pass filtering (0.01–0.1 Hz), movement correction by the algorithm of Cui et al.^51^ for correction of low amplitude movement artefacts, interpolation of single artefact-laden channels, a Gaussian PCA kernel for correction of the global signal^52^ and finally a z-standardization for better comparisons between subjects. Data was averaged over trials with a 15 s baseline correction and linear detrend before data was extracted for the anticipation phase (0–8 s) and exposure phase (9–19 s).

Electrophysiological data was analyzed with Brain Vision Analyser 2.1 software. EEG data was resampled at 256 Hz, re-referenced to the averaged mastoids, filtered with a butterworth zero phase bandpass with a low cut-off at 0.01 Hz (time constant 15.91549, 12 dB/oct) and a high cut-off at 14 Hz, 12 dB/oct. Ocular artefacts were corrected by using the HEOG/VEOG data with the ICA based ocular correction. We selected a rather low high cut-off filter, as we were primarily interested in the LPP which is driven by low frequency changes^53^. Further, data was segmented (−500 to 6,000 ms) for experimental triggers at the anticipation and confrontation phase with a 500 ms baseline correction. Before averaging the data to gain event-related potentials (ERPs), segments were checked for artefacts.

ECG data was filtered with a bandpass filter with a low cut-off at 5 Hz (time constant 0.03183099, 48 dB/oct) and a high cut-off at 45 Hz (48 dB/oct) before marker positions for R-peaks were detected with the pulse artefact correction tool and exported for each trigger-segment (anticipation and confrontation phase). Afterwards, the beats per minute were computed by summing up the R-peaks and dividing them by the total time of the concatenated segments of each condition.

EMG data was filtered with a bandpass filter with a low cut-off at 0.01 Hz (time constant 15.91549, 48 dB/oct) and a high cut-off at 200 Hz (48 dB/oct) and an additional notch filter at 50 Hz. Afterwards, data was segmented for triggers and further sub-segmented in 1 s long segments, before a Fast-Fourier-Transformation was performed with a Hanning window of 10% and window variance correction. Data was further averaged and exported for the range between 20 and 100 Hz to extract muscle related power density. We decided to measure the power of the signal, as the power of a signal provides a monopolar measure of the (squared) amplitude of that signal in the selected frequency spectrum^54^.

### Data analysis

fNIRS data was analyzed for 5 regions of interest (ROI) that cover most of the CCN: the left and right inferior frontal gyrus (IFG), the left and right dorsolateral prefrontal cortex (DLPFC) and the superior parietal lobule (SPL). To reduce complexity, we directly analyzed the contrast of the phobic spider condition and the non-phobic control condition. Therefore, we computed a mixed analysis of variance (ANOVA) on the difference scores with the between-subject factor group (patients vs. controls) and the within-subject factors phase (anticipation phase vs. exposure phase) and ROI (right IFG vs. right DLPFC vs. left IFG vs. left DLPFC vs. SPL).

In the same way, we analyzed differences (contrast: spider condition-control condition) in the time windows 450–1,000 ms and 1,000–6,000 ms of the ERP data. Note that we selected the time windows as we sought to measure the LPP. As we analyzed dynamic video stimuli, we expected to have limited power to measure small amplitude components and we expected a jittering of the latencies. Therefore, we limited the analysis to the LPP. To limit the number of analysis comparisons, we chose to analyze two time windows: an early LPP and late LPP phase. The definition of the early LPP time window was a compromise between the total time length of the used time frame in this study and definitions selected in previous ERP studies^12,53^. We conducted a mixed ANOVA with the factors group (patients vs. controls), caudal region (frontal vs. midline vs. parietal), hemisphere (left vs. midline vs. right) and phase (anticipation vs. exposure phase) separately for both time windows. Note that one subject was excluded from the ERP analysis as their contrast values were identified as outliers in most of the electrodes and conditions (> 3 SD within the phobic group of subjects). ECG and EMG data were also analyzed with mixed ANOVAs with the factors group (patients vs. controls) and phase (anticipation vs. exposure) for difference scores (contrast: spider condition-control condition).

Behavioral ratings at the end of each trial were analyzed as means over the trials of each condition. We computed the contrast of the spider and control condition and analyzed the difference values in a MANOVA with the factors group (PP vs. HC) and the dependent variables emotional distress and avoidance.

As HC and PP differed in age—although only in 4 years on average—we checked the analysis of fNIRS and EEG data using ANCOVAs with the covariate age. All reported results were replicated in this analysis.

To investigate the interaction of the hemodynamic data acquired via fNIRS and the electrophysiological data acquired via EEG, we computed between-subject Pearson correlations and repeated measures correlation coefficients based on Bakdash and Marusich (2017)^67^. In contrast to Pearson correlations, this correlation technique does not assume independence of error between observations, which is clearly violated in our study, where each participant provides multiple data points assessing EEG and fNIRS simultaneously. The r package rmcorr (Bakdash and Marusich 2017) uses parallel regression lines with varying intercepts, which are estimated using ANCOVA, to fit each data point^67^. In this manner, it can determine the relationship between two continuous variables. Assumptions required for rmcorr are quite similar to those for Generalized Linear Models, including the predictor´s linearity, independent and identically distributed and normally distributed errors. Furthermore, rmcorr assumes the slopes be parallel across conditions. All of these assumptions can be seen as fulfilled in our study design. We computed correlations between fNIRS ROIs and corresponding electrode positions. Note that we restricted the analysis to this ROI to electrode distribution to reduce the number of comparisons, despite the fact that EEG components might show maxima on other electrode positions. However, as the study of fNIRS-EEG measurements is just emerging, we used this analysis strategy as a starting point. Correction for multiple comparisons for the correlation analysis was done with the Benjamini–Hochberg procedure.

Finally, we performed a Discriminant Analysis to investigate which physiological variables predicted group membership the best. To limit available data, we used predictors at the level of significance of the prior analysis. For example, we used the ECG contrast of the grand mean, as the group variable showed a main effect for the ECG data.

All figures were created using the package “ggplot2” in R^55^. Brain maps were created with self-written code in MATLAB 2017 (a).

### Statement of ethics

The ethics committee at the University Hospital and University of Tuebingen approved this project and all subjects gave written informed consent. All methods and procedures used in this study were in accordance with current guidelines of the World Medical Association Declaration of Helsinki.

## Results

### Questionnaires and behavioral avoidance test

As expected, the groups differed significantly in the questionnaire measures of spider phobia and in the behavioral avoidance test (see Table 1). PP showed higher levels of spider phobia symptoms and spider phobia-related beliefs than HC. Furthermore, all control subjects were able to touch the spider by hand and let the spider walk over their arm. In contrast, 84% of the patients were not able to watch the spider from a distance closer than 2 m and none of the patients were able to touch a spider by hand. In summary, PP showed a consistent pattern of elevated behavioral, cognitive and emotional phobic symptoms in comparison to HC.

### Behavioral data

With respect to subjective ratings after each trial of watching videos, we observed a significant main effect of group [*F*(2, 59) = 63.356, Λ = .318, *p* < 0.001, *η*_*p*_^2^ = 0.68] indicating higher ratings in PP than in HC. Univariate analysis showed a significantly higher contrast in PP than in HC in anxiety [*F*(1, 60) = 128.858, p < .001, η_p_^2^ = .68] and avoidance ratings [*F*(1, 60) = 80.645, p < .001, η_p_^2^ = .57]. In summary, PP showed generally higher ratings than HC during the spider condition versus the control condition in avoidance and emotional distress (anxiety: PP: mean = 4.1, SD = 1.66; HC: mean = 0.35, SD = 0.56; avoidance: PP: mean = 3.6, SD = 1.88; HC: mean = 0.25, SD = 0.53).

### ECG

**Figure 2:**
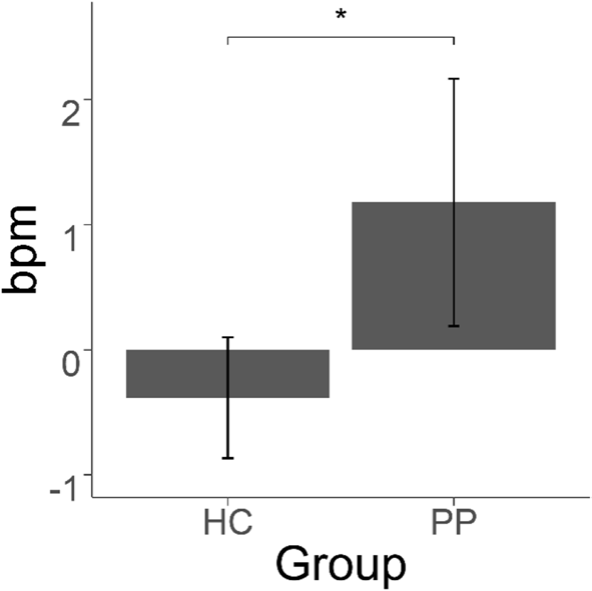
Contrast (spider-control condition) in heart rate between PP and HC. *p < 0.05.

The analysis of heart rate data (contrast: spider-control) showed a main effect of group [*F*(1, 58) = 6.300, *p* < 0.05, *η*_*p*_^2^ = 0.098], indicating higher heart rates in patients in the spider condition (*M* = 80.30) compared to the control condition (*M* = 79.12), with healthy subjects showing no significant changes in the spider condition (*M* = 73.17) versus the control condition (*M* = 73.56) (see figure of the contrast in Fig. 2).

### EMG

**Figure 3:**
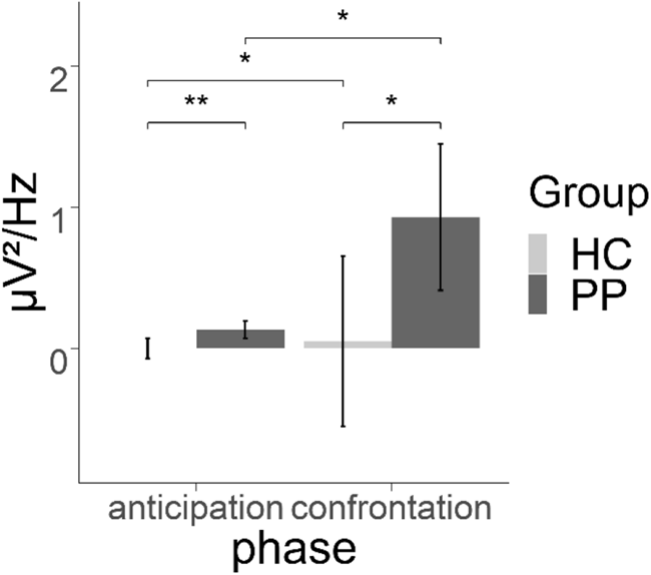
Difference in the contrast (spider-control) in EMG power in the anticipation and confrontation phase. *p < 0.05, **p < 0.01.

As hypothesized, when analyzing contrasts of EMG power (spider—control) we observed a significant constant term [*F*(1, 59) = 6.629, *p* < 0.05, *η*_*p*_^2^ = 0.10], reflecting higher EMG power during spider trials in comparison to control trials, and a significant main effect of group [*F*(1, 59) = 5.463, *p* < 0.05, *η*_*p*_^2^ = 0.09], reflecting a higher effect contrast in the patients compared to healthy subjects. Further, the main effect of phase [*F*(1,59) = 5.351, *p* < 0.05, *η*_*p*_^2^ = 0.08] and the interaction of group by phase [*F*(1, 59) = 4.133, *p* < 0.05, *η*_*p*_^2^ = 0.07] were significant, indicating a higher contrast during the confrontation phase than during the anticipation phase (main effect). The interaction was further analyzed by linear contrasts that revealed a stronger increase from the anticipation phase to the confrontation phase in the PP compared to the HC (see Fig. 3). In summary, PP showed increased EMG power during confrontation reflecting emotion-related facial expressions in the contrast of spider vs. control trials in comparison to the HC.

### fNIRS data

**Figure 4:**
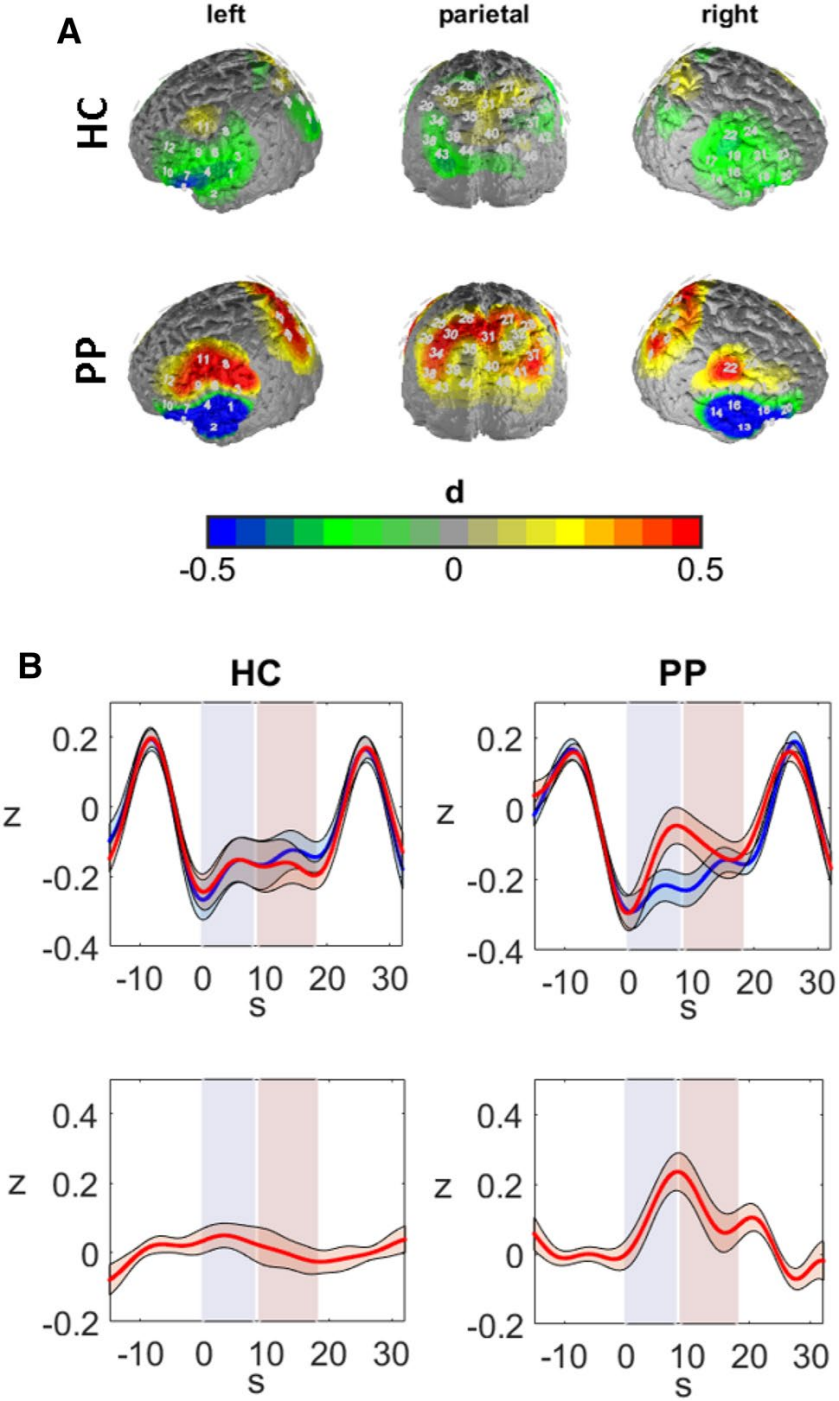
Effects in fNIRS data. (**A**) Brain maps of the contrast during the anticipation phase. Warm colors reflect higher O_2_Hb levels during the spider condition in comparison to the control condition. Differences are plotted in effect size Cohen’s d. (**B**) Hemodynamic response of the mean of DLPFC and SPL in HC (left) and PP (right) in the control condition (blue line) and spider condition (red line) (upper images) and the contrast (spider-control) (lower images). Horizontal shadings in blue mark the anticipation phase, red shadings mark the confrontation phase. Shadings around the hemodynamic curves reflect standard errors of the mean. Note that the huge hemodynamic responses at the end/beginning of each trial are due to the behavioral ratings. Therefore, O_2_Hb levels at the start of each trial were negative as the baseline included the 15 s before each trial; 0 s on the x-axis marks the beginning of the trial. The scaling represents z-standardized scores.

With respect to fNIRS data, we observed a significant interaction of group by phase by ROI [*F*(4, 212) = 3.560, p < 0.05, η_p_^2^ = 0.06]. The effect reflected increased O_2_Hb levels in the patients compared to the controls during the anticipation phase in the left IFG [*t*(53) = 2.918, *p* < 0.01, *d* = 0.79] and during the exposure phase in the SPL [*t*(53) = 2.510, *p* < 0.05, *d* = 0.66] (see Fig. 4).

Further, we observed a significant effect of ROI [*F*(4, 212) = 7.181, *p* < 0.001, *η*_*p*_^2^ = 0.12] and a marginally significant effect of group [*F*(1, 53) = 3.378, *p* < 0.1, *η*_*p*_^2^ = 0.06]. As we analyzed differences of the contrast (spider condition–control condition), these main effects indicate that there were significant differences between the ROI in the experimental conditions. Post-hoc analysis of comparisons against the mean of all ROI indicated that the experimental contrast was highest in the SPL [*t*(54) = 3.757, *p* < 0.001, *d* = 0.45] and lowest in the right DLPFC [*t*(54) = − 3.388, *p* < 0.001, *d* =  − 0.50]. The marginal main effect of group indicated a trend towards a generally higher experimental contrast in the patient group as compared to the control group.

In summary, PP showed elevated hemodynamic responses in different areas of the CCN dependent on the anticipation or actual confrontation with phobic stimuli in comparison to the HC.

### EEG data—ERP analysis

**Figure 5:**
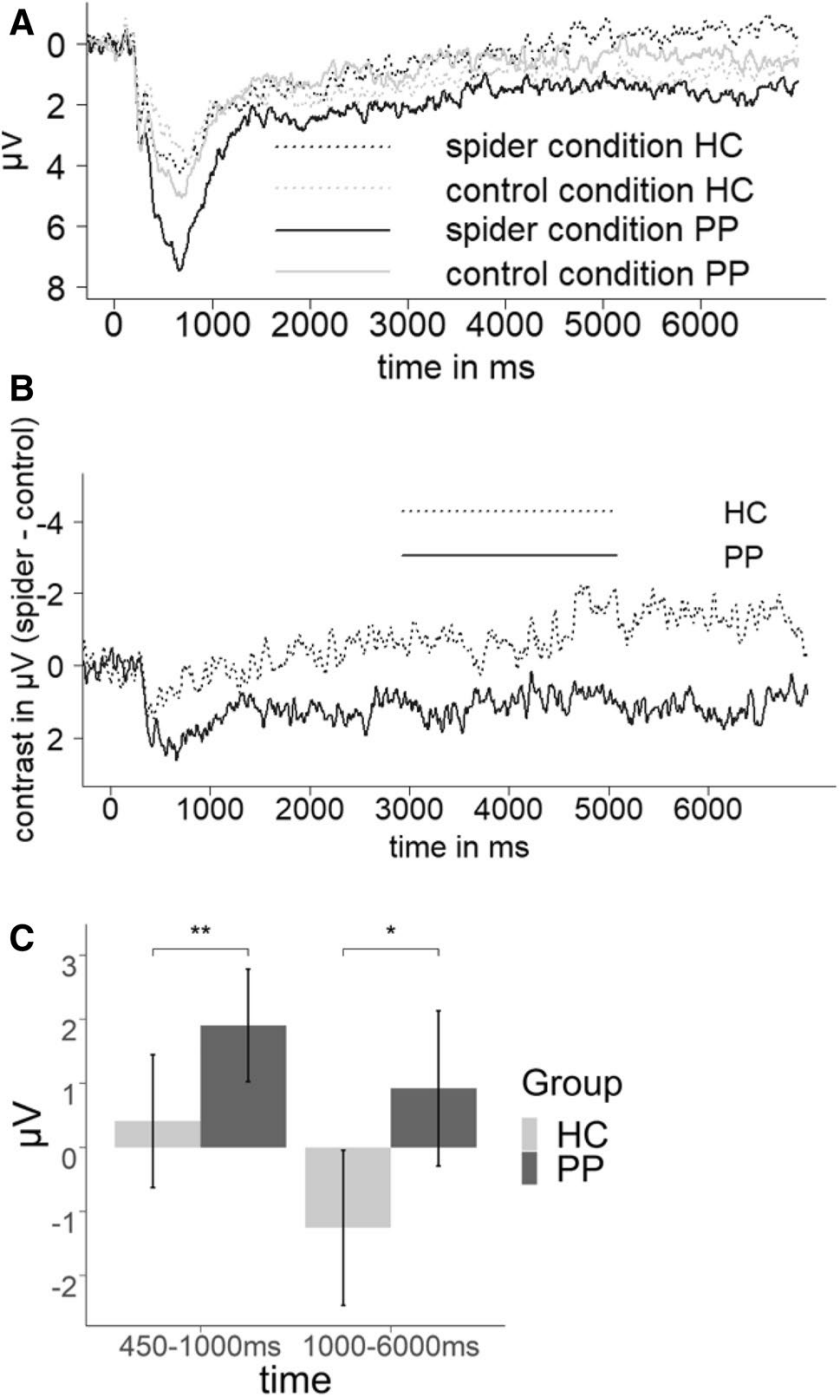
ERPs of the (**A**) experimental conditions as the mean of the anticipation and confrontation phase for HC and PP and (**B**) as the contrast for HC and PP. (**C**) Bar plots of the group main effect of the contrast (spider-control) during the early and late LPPs. Error bars indicate 95% confidence intervals. *p < 0.05, **p < 0.01.

With respect to ERP analysis during the late positive potential (450–1,000 ms), we observed a significant constant, reflecting a significant contrast [*F*(1, 58) = 7.448, *p* < 0.01, *η*_*p*_^2^ = 0.11] with a higher LPP in the spider condition than in the control condition. Further, we found a significant main effect of group [*F*(1, 57) = 8.240, *p* < 0.01, *η*_*p*_^2^ = 0.12] reflecting a higher contrast (higher LPP in the spider condition in comparison to control condition) in patients versus controls (see Fig. 5). Further, we observed a marginally significant interaction of caudal region by group [*F*(2, 114) = 2.598, *p* < 0.1, *η*_*p*_^2^ = 0.04] reflecting an increased effect in patients from anterior to posterior electrodes. We observed an additional interaction of hemisphere by phase, an effect unrelated to our research hypothesis on differences between patients and healthy controls [*F*(1.92, 111.66) = 3.233, *p* < 0.05, *η*_*p*_^2^ = 0.05]. Post-hoc analysis of linear contrasts revealed that the effect increased linearly from the anticipation to the exposure phase from the left to right hemisphere [*F*(1, 58) = 4.252, *p* < 0.05, *η*_*p*_^2^ = 0.07].

**Figure 6:**
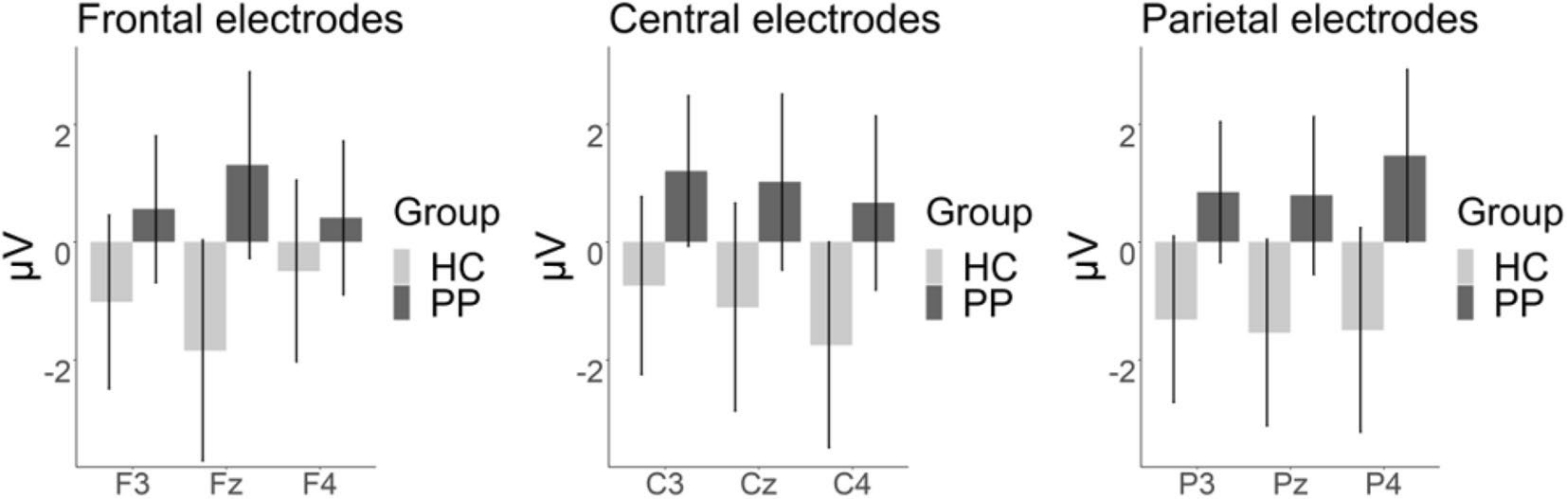
Mean values of the contrast (spider-control) LPP amplitudes for the interaction group*anterior region*hemisphere. Error bars indicate 95% confidence intervals.

In the very late timespan from 1,000 to 6,000 ms we also observed a significant main effect of group [*F*(1, 57) = 4.301, *p* < 0.05, *η*_*p*_^2^ = 0.07] reflecting a higher contrast for the patients than for the controls. Furthermore, we observed an interaction of group by caudal region by hemisphere [*F*(3.39, 196.75) = 3.208, *p* < 0.05, *η*_*p*_^2^ = 0.05]. Post-hoc analysis of polynomial contrasts revealed that the effect between patients and healthy controls increased from frontal to parietal electrodes and was highest in central regions (compared to left and right hemisphere) as indicated by a linear by quadratic interaction of caudal region by hemisphere [*F*(1, 57) = 5.532, *p* < 0.05, *η*_*p*_^2^ = 0.09] (see Fig. 6).

In summary, we observed elevated P300 and LPP components in the PP in comparison to the HC for the experimental contrast, with the latter effect showing a maximum at postcentral electrode sides.

### Correlation of fNIRS and ERP data

In an exploratory analysis we further examined the association between the EEG and fNIRS signals. To this end, we computed within-subject correlations with repeated measurement correlations and between-subject correlations between the experimental contrast values (spider condition–control condition) of the EEG and fNIRS signal. We used contrast-values for two reasons: First, we wanted to reduce the number of comparisons. Second, as both EEG and fNIRS are relative signals (EEG: to reference electrode, fNIRS: to baseline) we aimed to standardize the measurements between subjects by subtracting the control condition. To further minimize comparisons, we only compared the within-subject correlations of the ROI and corresponding electrode positions that are covered by the ROI. For between-subject comparisons we restricted our analysis to correlations between the level of significance of the above reported analysis. We compared the contrast values of phase and ROI of the fNIRS signal with the overall mean of the contrast of the EEG within the early and late time window (regardless of phase) and for each electrode position for the late time window (see below). Multiple comparisons were adjusted by the Benjamini–Hochberg procedure and results are reported for corrected and uncorrected comparisons.

The analysis of correlations between the fNIRS and EEG signal on a within-subject level revealed only significant positive correlations in the late time window of 1,000–6,000 ms when p values were corrected for multiple comparisons. We observed a significant within-subject correlation in the right ventrolateral prefrontal cortex [*r*(161) = 0.24, p < 0.01]. During the early time window (450–1,000 ms) we observed tendencies when not correcting for multiple comparisons within the parietal cortex [*r*(161) = 0.18, p < 0.05] and right ventrolateral prefrontal cortex [*r*(161) = 0.15, p < 0.1] (see Table 2).

**Table 2:** Repeated measurement correlations of fNIRS and EEG data.

	*r*	*df*	*p*	Significance
**450–1,000 ms**				
lIFG and mean of F3, Fz, C3, Cz	−0.000	161	0.990	n.s
lDLPFC and mean of F3, Fz, C3, Cz	−0.101	161	0.198	n.s
rIFG and mean of F4, Fz, C4, Cz	0.137	161	0.081	^†^
rDLPFC and mean of F4, Fz, C4, Cz	−0.071	161	0.365	n.s
SPL and mean of P3, Pz, P4	0.184	161	0.019	^†^
**1,000–6,000 ms**				
lIFG and mean of F3, Fz, C3, Cz	0.062	161	0.432	n.s
lDLPFC and mean of F3, Fz, C3, Cz	−0.072	161	0.364	n.s
rIFG and mean of F4, Fz, C4, Cz	0.244	161	0.002	*
rDLPFC and mean of F4, Fz, C4, Cz	−0.001	161	0.994	n.s
SPL and mean of P3, Pz, P4	0.102	161	0.197	n.s

EEG data was averaged over different electrodes to match the fNIRS regions of interest. Note that the correlational analysis was done including all experimental conditions: anticipation and confrontation phase, control and experimental condition. ^†^Significant or marginally significant without correction for multiple comparisons, *significant after correction for multiple comparisons by the Benjamini–Hochberg procedure.

On a between-subject level, even with a selection of a limited number of comparisons, the correction for multiple comparisons required a significance level of p < 0.0003 for the highest significant result. None of the between-subject level correlations survived this threshold. Tendencies were observed towards positive correlations between prefrontal ROIs (DLPFC and IFG) during anticipation (0.28 < *r* < 0.35, p_uncorr_ < 0.05) and LPP amplitudes in the grand average (all electrodes) in both time windows (450–1,000 ms and 1,000–6,000 ms). In the later time window, tendencies towards positive correlations (0.27 < *r* < 0.40, p_uncorr_ < 0.05) were predominantly present between the IFG in the anticipation phase and LPP amplitudes in the posterior electrodes (P3, Pz, P4) (see Table 3).

**Table 3:** Between-subject contrast (spider-control) correlations.

	lIFG P1	lDLPFC P1	rIFG P1	rDLPFC P1	SPL P1	lIFG P2	lDLPFC P2	rIFG P2	rDLPFC P2	SPL P2	R 1	R 2
GA 0.45-1s	0.267	0.215	0.347*	0.293*	0.006	0.083	− 0.001	0.151	0.035	0.027	0.342**	0.403**
GA 1s-6s	0.280*	0.229	0.335*	0.259	0.125	0.105	0.146	0.271*	0.183	0.193	0.341**	0.397**
F3 1s-6s	0.217	0.117	0.249	0.204	− 0.06	− 0.02	− 0.155	− 0.01	− 0.147	− 0.111	0.315*	0.354**
Fz 1s-6s	0.169	0.150	0.246	0.273*	− 0.03	0.114	− 0.004	0.105	0.056	− 0.06	0.271*	0.310*
F4 1s-6s	0.116	0.113	0.266	0.166	− 0.08	− 0.38	− 0.050	0.140	− 0.010	− 0.04	0.323*	0.347**
C3 1s-6s	0.211	0.177	0.275*	0.255	− 0.08	0.030	− 0.026	0.091	0.020	− 0.01	0.269*	0.349**
Cz 1s-6s	0.210	0.138	0.265	0.211	− 0.12	0.090	− 0.024	0.080	− 0.051	− 0.12	0.169	0.241
C4 1s-6s	0.273*	0.213	0.338*	0.261	0.020	0.202	0.162	0.250	0.202	0.127	0.273*	0.394**
P3 1s-6s	0.296*	0.261	0.406**	0.344*	0.131	0.081	0.032	0.189	0.088	0.158	0.362**	0.414**
Pz 1s-6s	0.325*	0.252	0.384**	0.313*	0.124	0.033	− 0.063	0.131	− 0.013	0.099	0.358**	0.399**
P4 1s-6s	0.348*	0.317*	0.377**	0.335*	0.143	0.188	0.143	0.267	0.158	0.208	0.392**	0.424**
R1	0.351**	0.196	0.338*	0.166	0.197	0.151	0.205	0.242	0.134	0.384**	1	0.914***
R2	0.351**	0.189	0.364**	0.198	0.209	0.153	0.179	0.241	0.162	0.389**	0.914***	1

*P1 =* anticipation phase, *P2* = exposure phase, *GA* = grand average.

*Note that p values were not significant after correction for multiple comparisons. p_uncorr_ < 0.05, **p_uncorr_ < 0.01.

### Discriminant analysis

In the exploratory discriminant analysis the following predictors were included: (1) contrast ECG grand mean, (2) contrast EMG during exposure, (3) contrast NIRS during anticipation in the left IFG and (4) during exposure in the SPL, (5) contrast EEG grand mean for 450–1,000 ms and (6) for 1,000–6,000 ms. Discriminant Analysis was conducted with the stepwise method. In the first step, the fNIRS contrast in the left IFG during anticipation was included in the model [F(1, 51) = 11.231, p < 0.01, Wilks Λ = 0.820]. In the second step, the contrast of EMG activity during exposure was included [F(2, 50) = 9.078, p < 0.001, Wilks Λ = 0.734]. All other variables were excluded. The function predicted in total 75.9% of group membership correctly with a sensitivity of 83% and specificity of 70%. In detail, the fNIRS contrast data correctly classified 67.3% and considering the EMG data improved the classification by 8.6%.

## Discussion

The aim of the study at hand was to investigate the neuronal correlates of spider-specific stimulus processing on an electrophysiological and hemodynamic level. As expected, spider phobic patients (PP) showed stronger late-positive potentials (LPP) and higher hemodynamic responses to phobia-related vs. neutral information in comparison to a control condition. Moreover, physiological correlates of emotional arousal by means of heart rate and facial expression measured by electromyography (EMG) showed consistent effects. Behaviorally, subjective ratings of emotional distress and avoidance were increased in PP in comparison to HC.

To our knowledge, this is the first study that investigates the neuronal correlates of PP in a simultaneous fNIRS-EEG study. First of all, our results showed that the paradigm used induced a phobic reaction consistent with reactions reported in the literature with increased subjective distress, avoidance^10,15,16,56^, heart rate^36^ and EMG-measured facial expressions^6,57,58^, indicating that videos are realistic enough to elicit a fear response in PP. Our results with respect to ERPs are well in line with the existing literature. In previous studies, elevated P300 and LPP amplitudes have been observed in PP when confronted with phobic stimuli^8,9,11,12^. It has been suggested that the effect of psychotherapy might not influence early ERPs of attention allocation and attentional significance, but rather late potentials of cognitive control. For example, the P300 has been shown to remain elevated after successful treatment, while increased LPPs after successful treatment have been observed^6,12^. Interestingly, we did not observe a difference in ERP amplitudes between the anticipation phase and confrontation phase, implying similar effects on the LPP for both anticipation of fearful objects, as well as the actual confrontation. This result is not surprising as subjects might already prepare for the confrontation with the fearful object during their anticipation^38^. Increased attentional allocation is in line with the model of increased post-encounter defense^5^. Similar results have been observed in a study of Michalski et al.^59^, who found increased LPPs in response to phobia-related pictures and cues in PP, which is interpreted as selective attention. Interestingly, so far, no video material has been used in EEG research on the matter of spider phobia, as video material is complex and might be a suboptimal starting point for the analysis of neuronal correlates of phobic information processing. However, with respect to symptom provocation, video material might be more plastic and realistic. Despite the complex nature of the video clips, our results regarding ERP components complement those evoked by static visual material.

With respect to hemodynamic changes, we observed a pattern of increased experimental contrast in PP but not in HC during anticipation in the left IFG and during confrontation in the SPL, reflecting higher hemodynamic responses to phobia-relevant information in spider phobics. Previous neuroimaging studies showed increased activity in limbic and prefrontal areas in PP during confrontation with phobic material^8,60–63^. Our results bolster this research and go on to show that the hemodynamic response already begins with the anticipatory regulation of the phobic stimulus^60,64^. Interestingly, the post-hoc analysis revealed that during anticipation prefrontal areas such as the IFG and DLPFC were predominantly active, while in the SPL differences between HC and PP were highest during confrontation. This data suggests a differential involvement of anterior and parietal cortex areas in the anticipation and actual confrontation with phobic stimuli. Indeed, from data in animal models and human neuroimaging studies, it has been hypothesized that prefrontal areas are involved in voluntary preparation and top-down control of anxiety^65,66^. In contrast, the invariably increased activity during the actual confrontation phase in the SPL most likely reflects increased attention allocation on—and visual processing of—the phobic stimuli within the PP^6,10,66^. Correspondingly, when looking at the relationship between the fNIRS, EEG and behavioral data we observed positive relationships between the three measures on a within-subject as well as on a between-subject level. However, as we had many multiple comparisons at this analysis step, only one comparison at the within-subject correlation and no comparison at the between-subject correlations were significant after correction by the Benjamini–Hochberg procedure. Tendencies towards positive associations between behavioral, EEG and fNIRS data were especially present in the SPL and IFG. The tendency towards positive correlation of the data within these regions is further supported by research of Scharmüller et al.^66^ who localized the LPP during confrontation in PP within the paracentral region, superior and medial regions, precuneus, as well as the insula, among other regions. In line with this localization, differences between HC and PP in the fNIRS data were observed within the IFG and SPL. On a behavioral level, increased LPPs and hemodynamic responses were associated with emotional distress and an urge to avoid the situation. At this point, it remains inconclusive if the increased CCN activity and LPP amlitudes might be seen as part of a flight or fight response during anticipation and confrontation with phobic stimuli. Some authors (Scharmüller et al.^66^) did suggest that cortical activity might reflect increased preparation of a flight reaction. However, it might also be possible that the increased prefrontal activity reflects cognitive control processes to endure the situation and inhibit active defense behavior as indicated by the activity in the IFG^27^. In line with this theory, increased LPP potentials were found after successful CBT treatment of spider phobia^6,12^. On the other hand, in recently published data from our group, we found a decreased hemodynamic response in the CCN within consecutive sessions of exposure therapy^38^. However, we interpreted these decreases in CCN activity within the scope of a scaffolding network that is active at the beginning of a psychotherapy session and declines with the ongoing session. Lastly, it might be the case that both interpretations are valid, because different behavioral responses are primed and prepared unconsciously during a confrontation with fearful material. In future studies, it might be interesting to allow subjects to flee from such a situation, e.g. by closing their eyes or physically leaving the room, which is possible with wearable EEG and fNIRS devices. Within this study, we show that multimodal measurements (fNIRS–EEG–EMG–ECG) are a convenient method to further describe brain mechanisms involved in the processing of imminence of threat. This is highly relevant to a better understanding of anxiety disorders and may allow new treatment approaches to be developed.

Finally, our exploratory discriminant analysis showed that NIRS data during the anticipation phase and EMG data during the exposure phase together discriminated between the subject groups at best, while the other variables have been excluded. While this result to some extent underlines the sensitivity of the fNIRS and EMG data, it is important to recognize that this analysis step was just an exploratory one and the selection of the predictors was mostly data-driven by the previously performed analysis. Although EEG data and ECG data showed significant results in the analysis, their explanation in the differentiation between healthy and phobic participants seems to be already and best captured by the fNIRS and EMG data.

Despite these promising findings, some limitations have to be considered. Although the combination of neuroimaging methods has some advantages, like complementary information gleaned from their respective strengths, it is important to bear in mind that it also merges the weaknesses of both procedures. For example, EEG has a very high temporal resolution, while the hemodynamic response is incredibly slow in relation to neuronal firing of cell assemblies. Therefore, it is not totally conclusive how EEG components and hemodynamic responses can be interpreted within the time-domain aside from localizing the low spatial resolution of the ERP components more accurately, a technique which is yet to be optimized. The mechanism of how a P300 or LPP amplitude is related to a hemodynamic response that might occur seconds later cannot be concluded from multimethod studies in humans per se. It may be the case that future invasive animal studies might be able to answer these questions. Furthermore, fNIRS measurement is restricted to the upper parts of the cortex, as the penetration depth of the near-infrared light is limited to approximately 3 cm. In the current study, we used the fNIRS method for reasons of ecological validity and cost effectiveness. In future studies, the results of this analysis might be extended to fMRI–EEG measurements or even fMRI–EEG–fNIRS measurements. However, these study designs might have their own merits and limitations. Lastly, in future investigations it might be beneficial to include further conditions in which, e.g. dangerous but not phobia-related animals (e.g. tigers vs. spiders) are presented.

## Conclusions

This is the first study that combined fNIRS and EEG measurements in the study of specific phobia. Further, this is the first study that uses dynamic instead of static stimulus material. In line with existing research, we observed increased LPP amplitudes and hemodynamic responses in spider phobic patients in response to phobia-related information. Subjective ratings, fNIRS and EEG data were positively associated on a within- and between-subject level, indicating a valid measurement in all three assessments.

## Supplementary information

Supplementary file1
