# Supplementary material for: Neuronal correlates of spider phobia in a combined fNIRS-EEG study
Source: Sci Rep. 2020 Jul 28;10:12597. doi: 10.1038/s41598-020-69127-3 (PMC7387441; doi:10.1038/s41598-020-69127-3)

**Supplementary Material: Neuronal Correlates of Spider Phobia in a combined fNIRS-EEG Study**

David Rosenbaum^1^, Elisabeth J. Leehr^2^, Agnes Kroczek^1^, Julian A. Rubel^3^, Isabell Int-Veen^1^, Kira Deutsch^1^, Moritz J. Maier^1^, Justin Hudak^4^, Andreas J. Fallgatter^1,5^, Ann-Christine Ehlis^1,5^

^1^ Department of Psychiatry and Psychotherapy, University Hospital of Tuebingen, Tuebingen, Germany

# ^2^ Department of Psychiatry, University of Muenster, Muenster, Germany

^3^ Department of Psychotherapy Research, Justus-Liebig-University Giessen, Giessen, Germany

^4^ Center on Mindfulness and Integrative Health Intervention Development, University of Utah, Salt Lake City, UT 84112, USA

^5^ LEAD Graduate School & Research Network, University of Tuebingen, Tuebingen, Germany

**spider videos**

video 1: Close-up of an Argiope bruennichi emitting silk and building a web in a forest.

video 2: Close-up of an Argiope bruennichi abseiling in a forest.

video 3: Close-up of an Argiope bruennichi from below walking on her web in a forest.

video 4: Close-up of spider webs glistening with dew, the last one with a Tegenaria domestica in a forest.

video 5: Zoom-in on Tegenaria domestica resting in her spider web and Close-up of her from below walking on her web in a forest.

video 6: Close-up of a Tegenaria domestica taking down her own web in a forest.

video 7: Pan shot of a spider web and Close-up of a non-moving Araneus diadematus in a forest.

video 8: Pan shot of a spider web and Close-up of a non-moving Pholcus phalangioides in a forest.

video 9: Pan shot and Close-Up of a spotlight searching for a Eratigena atrica in a shed.

video 10: Pan shot of a dusty spider web outside a shed and Close-up of a Scotophaeus blackwalli catching a fly.

video 11: Super Close-Up of a Scotophaeus blackwalli eating a fly.

video 12: Super Close-Up of a Scotophaeus blackwalli eating a fly and Zoom-Out.

video 13: Zoom-Out of a non-moving Scotophaeus blackwalli. Close-Up of a man regarding a Pholcus opilionoides.

video 14: Close-Up of a Pholcus opilionoides swinging her web.

video 15: Close-Up of a Pholcus opilionoides and a Badumna insignis swinging her web.

video 16: Close-Up of a Pholcus opilionoides swinging her web outside.

video 17: Close-Up of a Alopecosa catching a fly outside.

video 18: Close-Up of a Alopecosa abseiling in a forest.

video 19: Close-Up of a Scytodes thoracica moving on a rock in the dark.

video 20: Close-Up of a Scytodes thoracica moving on a rock in the dark.

video 21: Close-Up of a Scytodes thoracica moving under a shelf. Establishing shot and Zoom-In of a man walking towards the shelf.

video 22: Close-Up of a Misumena vatia eating a butterfly.

video 23: Close-Up of a Pardosa amentata eating a grasshopper.

video 24: Close-Ups of various spiders eating grasshoppers.

video 25: Close-Up of a Nuctenea umbratica on a leaf feeling its way.

video 26: Close-Up of a Nuctenea umbratica on a leaf feeling its way.

video 27: Close-Up and slow motion of Nuctenea umbratica on a leaf feeling its way.

video 28: Close-Up of a Nuctenea umbratica on a leaf feeling its way.

video 29: Close-Up of a Nuctenea umbratica on a leaf feeling its way.

video 30: Close-Up of a Nuctenea umbratica on a leaf feeling its way.

video 31: Establishing shot of a Ozyptila praticola coming closer to another spider.

video 32: Establishing shot of a Ozyptila praticola coming closer to another spider.

video 33: Establishing shot of a Ozyptila praticola coming closer to another spider and raising their foremost legs.

video 34: Establishing shot of a Ozyptila praticola coming closer to another spider and raising their foremost legs and moving even closer.

video 35: Close-Up of a spider walking in the web of a giant spider.

video 36: Close-Up of a spider walking in the web of a giant spider.

video 37: Close-Up of a spider walking on a giant spider.

video 38: Close-Up of a spider walking on a giant spider.

video 39: Close-Up of a spider walking on a giant spider.

video 40: Close-Up of a spider walking on a giant spider.

video 41: Close-Up of a spider walking on a giant spider.

video 42: Close-Up of a spider walking on a giant spider.

video 43: Close-Up of an Argiope bruennichi laying eggs.

video 44: Close-Up of an Argiope bruennichi laying eggs and weaving a layer of silk around them.

video 45: Super Close-Up of an Argiope bruennichi weaving a layer of silk around her eggs.

video 46: Super Close-Up of an Argiope bruennichi weaving a layer of silk around her eggs in fast motion.

video 47: Super Close-Up of an Argiope bruennichi weaving a layer of silk around her eggs in fast motion.

video 48: Close-Up of an Argiope bruennichi weaving a layer of silk around her eggs in fast motion.

video 49: Close-Up of an Argiope bruennichi weaving a layer of silk around her eggs in fast motion.

video 50: Close-Up of an Argiope bruennichi weaving a layer of silk around her eggs.

video 51: Close-Up of an Argiope bruennichi weaving a layer of silk around her eggs.

video 52: Close-Up of an Argiope bruennichi weaving a layer of silk around her eggs.

video 53: Close-Up of an Argiope bruennichi weaving a layer of silk around her eggs and leaving the cocoon with eggs.

video 54: Close-Up of an Argiope bruennichi leaving the cocoon with eggs. Slow Motion of a Pardosa amentata with egg cocoon jumping.

video 55: Close-Up of a Pardosa amentata carrying an egg walking.

video 56: Close-Up of a dead Pardosa amentata carrying an egg sac with baby spiderlings hatching from the eggs.

video 57: Close-Up of a dead Pardosa amentata carrying an egg sac with baby spiderlings hatching from the eggs.

video 58: Close-Up of a dead Pardosa amentata carrying an egg sac with baby spiderlings hatching from the eggs and moving over the dead body of their mother.

video 59: Close-Up of a dead Pardosa amentata carrying an egg sac with baby spiderlings hatching from the eggs and moving over the dead body of their mother. Close-Up of a spider running in fast and afterward slow motion.

video 60: Close-Up of a Neriene peltata and baby spiderlings hatching from eggs.

video 61: Super Close-Up of a Misumena vatia on a dead fly. Close-Up of a frog who is about to catch something.

video 62: Establishing shot of a non-moving Argiope bruennichi in a web in the forest.

video 63: Close-Up of insects getting stuck in a spider web.

video 64: Close-Up of insects getting stuck in a spider web and spiders wrapping their prey in silk.

video 65: Close-Up of insects getting stuck in a spider web and spiders wrapping their prey in silk.

**neutral videos**

video 1: Establishing shot of a Canis lupus on a lead sniffing out flesh in a cage.

video 2: Establishing shot and Close-Up of a Golden Retriever on a lead sniffing out flesh in a cage.

video 3: Close-Up of a Canis lupus on a lead sniffing out flesh in a cage.

video 4: Close-Up of a Canis lupus on a lead sniffing out flesh in a cage shaking it.

video 5: Close-Up of a Golden Retriever on a lead sniffing out flesh in a cage and trying to grab it.

video 6: Close-Up of a Golden Retriever looking at his master.

video 7: Close-Up of a Canis lupus shaking and a woman kissing him on his cheek.

video 8: Close-Up of two goats, a Golden Retriever puppy walking up and a mature Golden Retriever.

video 9: Close-Up and Zoom-In of a mature Golden Retriever and a sheep flock.

video 10: Pan shot of kitten of different cat breeds playing with each other.

video 11: Pan shot of kitten of different cat breeds playing with each other.

video 12: Close-Up of kitten of different cat breeds playing with ropes.

video 13: Close-Up of kitten of different cat breeds playing with ropes and a woman smiling at them laying under an infrared lamp.

video 14: Close-Up of kitten of different cat breeds playing with ropes and a woman smiling at them laying under an infrared lamp.

video 15: Close-Up of kittens of different cat breeds laying under an infrared lamp.

video 16: Close-Up of kittens of different cat breeds laying under an infrared lamp.

video 17: Establishing shots of European Shorthairs jumping to catch ropes.

video 18: Establishing shots and slow motion of European Shorthairs jumping to catch ropes.

video 19: Establishing shot and Close-Up of European Shorthairs jumping to catch ropes and playing.

video 20: Close-Up and Pan shot of European Shorthairs.

video 21: Close-Up of a women and a man both enjoying to pet their European Shorthairs.

video 22: Close-Up of a man enjoying to pet and play with his European Shorthair.

video 23: Close-Up of different cat breeds playing with feathers.

video 24: White Hovawart watching over a flock of sheep.

video 25: Two white Hovawart watching over a flock of sheep.

video 26: Establishing shot of two non-moving Beagles.

video 27: Pan shot and establishing shot of a Beagle carrying a duck-like dummy in his jaw crossing a river.

video 28: Pan shot of kitten of different cat breeds playing with each other.

video 29: Pan shot of kitten of different cat breeds playing with each other. Close-Up of a non-moving mature European Shorthair.

video 30: Pan shot of kitten of different cat breeds playing with a rope and each other.

video 31: Establishing shot of two women petting kittens of different cat breeds while they play.

video 32: Slow Motion establishing shot of two European Shorthairs playing.

video 33: Super Close-Up of cat eyes.

video 34: Super Close-Up of a cat's face.

video 35: Super Close-Up of a cat's face and a dog's nose.

video 36: Super Close-Up of a dog's nose and Close-Up of a Beagle's face.

video 37: Close-Up of an Abyssinian and a European Shorthair.

video 38: Close-Up of different non-moving kittens of different cat breeds.

video 39: Close-Up of different non-moving kittens of different cat breeds.

video 40: Close-Up of a sleeping European Shorthair and Super Close-Up of a cat's face.

video 41: Super Close-Up of a cat's face and fur.

video 42: Establishing shot of a European Shorthair sniffing out a box.

video 43: Establishing shot of a European Shorthair sniffing out a box.

video 44: Close-Up of dog's faces of different dog breeds.

video 45: Close-Up of dog's faces of different dog breeds.

video 46: Pan shot of bird's-eye view showing a sheep flock. Close-Up of a mature white Golden Retriever.

video 47: Close-Up of a mature white Golden Retriever and a man counting sheep.

video 48: Close-Up of a mature white Golden Retriever. Pan shot of two men talking.

video 49: Establishing shot of a Golden Retriever and a sheep flock.

video 50: Establishing shot of a Golden Retriever and a sheep flock.

video 51: Establishing shot and Close-Up of Establishing shot of a Golden Retriever and several sheep.

video 52: Establishing shot of a Golden Retriever and a sheep flock.

video 53: Zoom-Out of bird ́s-eye view showing a sheep flock. Close-Up of dog's noses.

video 54: Close-Up of a dog's face. Establishing shot of two puppies sniffing.

video 55: Establishing shot of two puppies sniffing and a women in uniform applying a dog lead on a German Shepherd.

video 56: Establishing shot of a women in uniform taking a German Shepherd in the lead.

video 57: Establishing shot of a women in uniform taking a German Shepherd in the lead.

video 58: Pan shot of a baby jaguar in a tree and a man smiling at him as he touches his shoulders.

video 59: Establishing shot of a man smiling at a baby jaguar as he touches his shoulders and explaining something to the camera.

video 60: Close-Up of a cheetah's face and a wildcat hunting in the snow.

video 61: Close-Up of a lion ́s and a cheetah's face.

video 62: Establishing shot of a tiger in the snow and a cheetah jumping.

video 63: Establishing shot of a cheetah jumping and a lion walking in the savannah.

Supplementary Tables: Ratings for spider and control videos, V = Version, HC = healthy controls, PP = phobic patients

| Video | | | | N | | min | | max | | mean | | SD | |
| --- | --- | --- | --- | --- | --- | --- | --- | --- | --- | --- | --- | --- | --- |
|  |  |  |  | HC | PP | HC | PP | HC | PP | HC | PP | HC | PP |
| 1 | Spider Videos | V1 | rating1 | 10 | 13 | 1 | 1 | 3 | 9 | 1,50 | 5,38 | 0,707 | 2,256 |
|  |  |  | rating2 | 10 | 13 | 1 | 1 | 3 | 8 | 1,20 | 5,15 | 0,632 | 2,478 |
|  |  | V2 | rating1 | 9 | 10 | 1 | 3 | 3 | 9 | 1,33 | 5,20 | 0,707 | 2,201 |
|  |  |  | rating2 | 9 | 10 | 1 | 1 | 2 | 9 | 1,22 | 4,10 | 0,441 | 2,644 |
|  |  | V3 | rating1 | 8 | 12 | 1 | 2 | 3 | 9 | 1,38 | 5,75 | 0,744 | 2,379 |
|  |  |  | rating2 | 8 | 12 | 1 | 2 | 2 | 9 | 1,25 | 5,67 | 0,463 | 2,060 |
| 2 | Spider Videos | V1 | rating1 | 10 | 13 | 1 | 1 | 3 | 8 | 1,60 | 5,00 | 0,843 | 2,121 |
|  |  |  | rating2 | 10 | 13 | 1 | 1 | 3 | 8 | 1,30 | 4,77 | 0,675 | 2,488 |
|  |  | V2 | rating1 | 9 | 10 | 1 | 1 | 3 | 9 | 1,67 | 5,00 | 0,866 | 2,404 |
|  |  |  | rating2 | 9 | 10 | 1 | 1 | 2 | 9 | 1,44 | 4,40 | 0,527 | 2,989 |
|  |  | V3 | rating1 | 8 | 12 | 1 | 3 | 2 | 9 | 1,38 | 6,83 | 0,518 | 2,125 |
|  |  |  | rating2 | 8 | 12 | 1 | 3 | 2 | 9 | 1,25 | 6,67 | 0,463 | 1,875 |
| 3 | Spider Videos | V1 | rating1 | 10 | 13 | 1 | 2 | 3 | 9 | 1,30 | 5,38 | 0,675 | 2,399 |
|  |  |  | rating2 | 10 | 13 | 1 | 1 | 2 | 9 | 1,10 | 5,15 | 0,316 | 2,794 |
|  |  | V2 | rating1 | 9 | 10 | 1 | 1 | 4 | 9 | 1,67 | 6,20 | 1,118 | 2,616 |
|  |  |  | rating2 | 9 | 10 | 1 | 1 | 3 | 9 | 1,44 | 5,20 | 0,882 | 3,293 |
|  |  | V3 | rating1 | 8 | 12 | 1 | 1 | 2 | 6 | 1,25 | 4,08 | 0,463 | 1,621 |
|  |  |  | rating2 | 8 | 12 | 1 | 1 | 1 | 6 | 1,00 | 3,42 | 0,000 | 1,443 |
| 4 | Spider Videos | V1 | rating1 | 10 | 13 | 1 | 1 | 5 | 6 | 1,40 | 3,38 | 1,265 | 1,805 |
|  |  |  | rating2 | 10 | 13 | 1 | 1 | 6 | 6 | 1,50 | 2,85 | 1,581 | 1,405 |
|  |  | V2 | rating1 | 9 | 10 | 1 | 3 | 4 | 9 | 1,67 | 5,90 | 1,118 | 2,183 |
|  |  |  | rating2 | 9 | 10 | 1 | 1 | 3 | 9 | 1,67 | 5,00 | 0,866 | 2,944 |
|  |  | V3 | rating1 | 8 | 12 | 1 | 3 | 2 | 7 | 1,13 | 4,92 | 0,354 | 1,505 |
|  |  |  | rating2 | 8 | 12 | 1 | 2 | 1 | 7 | 1,00 | 4,42 | 0,000 | 1,730 |
| 5 | Spider Videos | V1 | rating1 | 10 | 13 | 1 | 2 | 3 | 8 | 1,30 | 4,92 | 0,675 | 2,019 |
|  |  |  | rating2 | 10 | 13 | 1 | 1 | 4 | 8 | 1,30 | 4,54 | 0,949 | 2,106 |
|  |  | V2 | rating1 | 9 | 10 | 1 | 1 | 3 | 8 | 1,33 | 4,90 | 0,707 | 2,424 |
|  |  |  | rating2 | 9 | 10 | 1 | 1 | 3 | 8 | 1,44 | 4,20 | 0,882 | 2,700 |
|  |  | V3 | rating1 | 8 | 12 | 1 | 2 | 2 | 7 | 1,25 | 4,50 | 0,463 | 1,446 |
|  |  |  | rating2 | 8 | 12 | 1 | 2 | 2 | 7 | 1,13 | 3,83 | 0,354 | 1,586 |
| 6 | Spider Videos | V1 | rating1 | 10 | 13 | 1 | 1 | 2 | 8 | 1,30 | 4,62 | 0,483 | 2,181 |
|  |  |  | rating2 | 10 | 13 | 1 | 1 | 3 | 8 | 1,20 | 3,69 | 0,632 | 2,394 |
|  |  | V2 | rating1 | 9 | 10 | 1 | 1 | 3 | 8 | 1,33 | 4,40 | 0,707 | 2,319 |
|  |  |  | rating2 | 9 | 10 | 1 | 1 | 2 | 7 | 1,22 | 3,50 | 0,441 | 2,068 |
|  |  | V3 | rating1 | 8 | 12 | 1 | 1 | 2 | 8 | 1,25 | 4,42 | 0,463 | 1,975 |
|  |  |  | rating2 | 8 | 12 | 1 | 1 | 2 | 9 | 1,13 | 3,67 | 0,354 | 2,060 |

| Video | | | | N | | min | | max | | mean | | SD | |
| --- | --- | --- | --- | --- | --- | --- | --- | --- | --- | --- | --- | --- | --- |
|  |  |  |  | HC | PP | HC | PP | HC | PP | HC | PP | HC | PP |
| 7 | Spider Videos | V1 | rating1 | 10 | 13 | 1 | 2 | 4 | 9 | 1,60 | 6,46 | 1,075 | 2,145 |
|  |  |  | rating2 | 10 | 13 | 1 | 1 | 3 | 9 | 1,30 | 5,62 | 0,675 | 2,785 |
|  |  | V2 | rating1 | 9 | 10 | 1 | 2 | 3 | 7 | 1,44 | 4,70 | 0,882 | 1,767 |
|  |  |  | rating2 | 9 | 10 | 1 | 1 | 3 | 8 | 1,33 | 3,40 | 0,707 | 2,413 |
|  |  | V3 | rating1 | 8 | 12 | 1 | 2 | 2 | 8 | 1,13 | 4,17 | 0,354 | 2,167 |
|  |  |  | rating2 | 8 | 12 | 1 | 2 | 1 | 8 | 1,00 | 3,58 | 0,000 | 1,929 |
| 8 | Spider Videos | V1 | rating1 | 10 | 13 | 1 | 2 | 3 | 8 | 1,40 | 5,62 | 0,699 | 1,981 |
|  |  |  | rating2 | 10 | 13 | 1 | 1 | 2 | 8 | 1,10 | 5,00 | 0,316 | 2,449 |
|  |  | V2 | rating1 | 9 | 10 | 1 | 1 | 2 | 8 | 1,22 | 5,10 | 0,441 | 2,470 |
|  |  |  | rating2 | 9 | 10 | 1 | 1 | 2 | 8 | 1,11 | 4,00 | 0,333 | 2,708 |
|  |  | V3 | rating1 | 8 | 12 | 1 | 2 | 2 | 8 | 1,25 | 4,33 | 0,463 | 1,923 |
|  |  |  | rating2 | 8 | 12 | 1 | 2 | 2 | 8 | 1,25 | 3,58 | 0,463 | 1,832 |
| 9 | Spider Videos | V1 | rating1 | 10 | 13 | 1 | 3 | 4 | 9 | 1,80 | 6,77 | 1,033 | 2,127 |
|  |  |  | rating2 | 10 | 13 | 1 | 2 | 5 | 9 | 1,40 | 6,46 | 1,265 | 2,436 |
|  |  | V2 | rating1 | 9 | 10 | 1 | 3 | 4 | 7 | 1,44 | 5,10 | 1,014 | 1,663 |
|  |  |  | rating2 | 9 | 10 | 1 | 1 | 3 | 8 | 1,33 | 4,60 | 0,707 | 2,716 |
|  |  | V3 | rating1 | 8 | 12 | 1 | 2 | 2 | 8 | 1,25 | 4,08 | 0,463 | 1,881 |
|  |  |  | rating2 | 8 | 12 | 1 | 1 | 2 | 8 | 1,13 | 3,33 | 0,354 | 1,969 |
| 10 | Spider Videos | V1 | rating1 | 10 | 13 | 1 | 4 | 5 | 9 | 2,00 | 7,15 | 1,633 | 1,908 |
|  |  |  | rating2 | 10 | 13 | 1 | 3 | 4 | 9 | 1,40 | 7,31 | 0,966 | 1,797 |
|  |  | V2 | rating1 | 9 | 10 | 1 | 2 | 3 | 8 | 1,56 | 5,30 | 0,882 | 2,214 |
|  |  |  | rating2 | 9 | 10 | 1 | 1 | 3 | 8 | 1,33 | 4,30 | 0,707 | 2,584 |
|  |  | V3 | rating1 | 8 | 12 | 1 | 2 | 2 | 6 | 1,25 | 4,00 | 0,463 | 1,414 |
|  |  |  | rating2 | 8 | 12 | 1 | 2 | 3 | 7 | 1,25 | 3,50 | 0,707 | 1,679 |
| 11 | Spider Videos | V1 | rating1 | 10 | 13 | 1 | 2 | 5 | 9 | 2,50 | 6,15 | 1,269 | 2,230 |
|  |  |  | rating2 | 10 | 13 | 1 | 1 | 3 | 9 | 1,60 | 6,00 | 0,843 | 2,739 |
|  |  | V2 | rating1 | 9 | 10 | 1 | 1 | 3 | 9 | 1,33 | 3,60 | 0,707 | 2,757 |
|  |  |  | rating2 | 9 | 10 | 1 | 1 | 2 | 8 | 1,22 | 2,80 | 0,441 | 2,348 |
|  |  | V3 | rating1 | 8 | 12 | 1 | 1 | 2 | 6 | 1,13 | 3,67 | 0,354 | 1,497 |
|  |  |  | rating2 | 8 | 12 | 1 | 1 | 2 | 5 | 1,13 | 2,92 | 0,354 | 1,443 |
| 12 | Spider Videos | V1 | rating1 | 10 | 13 | 1 | 2 | 5 | 9 | 2,10 | 5,92 | 1,287 | 2,178 |
|  |  |  | rating2 | 10 | 13 | 1 | 2 | 4 | 9 | 1,60 | 6,08 | 0,966 | 2,499 |
|  |  | V2 | rating1 | 9 | 10 | 1 | 1 | 3 | 9 | 1,44 | 3,80 | 0,726 | 2,486 |
|  |  |  | rating2 | 9 | 10 | 1 | 1 | 3 | 8 | 1,22 | 2,90 | 0,667 | 2,558 |
|  |  | V3 | rating1 | 8 | 12 | 1 | 2 | 2 | 8 | 1,13 | 4,83 | 0,354 | 2,209 |
|  |  |  | rating2 | 8 | 12 | 1 | 1 | 2 | 8 | 1,13 | 3,92 | 0,354 | 2,314 |

| Video | | | | N | | min | | max | | mean | | SD | |
| --- | --- | --- | --- | --- | --- | --- | --- | --- | --- | --- | --- | --- | --- |
|  |  |  |  | HC | PP | HC | PP | HC | PP | HC | PP | HC | PP |
| 13 | Spider Videos | V1 | rating1 | 10 | 13 | 1 | 2 | 5 | 7 | 1,60 | 5,08 | 1,265 | 1,656 |
|  |  |  | rating2 | 10 | 13 | 1 | 1 | 2 | 7 | 1,20 | 4,92 | 0,422 | 1,656 |
|  |  | V2 | rating1 | 9 | 10 | 1 | 1 | 3 | 9 | 1,56 | 3,80 | 0,882 | 2,530 |
|  |  |  | rating2 | 9 | 10 | 1 | 1 | 3 | 9 | 1,33 | 3,10 | 0,707 | 2,767 |
|  |  | V3 | rating1 | 8 | 12 | 1 | 1 | 2 | 7 | 1,25 | 4,25 | 0,463 | 1,765 |
|  |  |  | rating2 | 8 | 12 | 1 | 1 | 2 | 6 | 1,13 | 3,50 | 0,354 | 1,784 |
| 14 | Spider Videos | V1 | rating1 | 10 | 13 | 1 | 2 | 3 | 7 | 1,20 | 5,00 | 0,632 | 1,871 |
|  |  |  | rating2 | 10 | 13 | 1 | 1 | 2 | 9 | 1,10 | 4,77 | 0,316 | 2,488 |
|  |  | V2 | rating1 | 9 | 10 | 1 | 1 | 3 | 9 | 1,44 | 4,20 | 0,726 | 3,048 |
|  |  |  | rating2 | 9 | 10 | 1 | 1 | 3 | 9 | 1,22 | 4,10 | 0,667 | 3,107 |
|  |  | V3 | rating1 | 8 | 12 | 1 | 3 | 2 | 8 | 1,25 | 5,42 | 0,463 | 1,975 |
|  |  |  | rating2 | 8 | 12 | 1 | 2 | 2 | 7 | 1,25 | 4,67 | 0,463 | 1,723 |
| 15 | Spider Videos | V1 | rating1 | 10 | 13 | 1 | 2 | 3 | 8 | 1,40 | 6,15 | 0,699 | 2,410 |
|  |  |  | rating2 | 10 | 13 | 1 | 1 | 2 | 9 | 1,10 | 5,85 | 0,316 | 2,734 |
|  |  | V2 | rating1 | 9 | 10 | 1 | 2 | 2 | 8 | 1,33 | 5,10 | 0,500 | 2,183 |
|  |  |  | rating2 | 9 | 10 | 1 | 1 | 4 | 8 | 1,33 | 4,20 | 1,000 | 2,616 |
|  |  | V3 | rating1 | 8 | 12 | 1 | 4 | 3 | 9 | 1,50 | 6,08 | 0,756 | 1,564 |
|  |  |  | rating2 | 8 | 12 | 1 | 4 | 2 | 9 | 1,25 | 5,75 | 0,463 | 1,485 |
| 16 | Spider Videos | V1 | rating1 | 10 | 13 | 1 | 2 | 3 | 8 | 1,40 | 5,08 | 0,699 | 1,977 |
|  |  |  | rating2 | 10 | 13 | 1 | 1 | 2 | 9 | 1,20 | 4,15 | 0,422 | 2,512 |
|  |  | V2 | rating1 | 9 | 10 | 1 | 2 | 3 | 8 | 1,56 | 5,00 | 0,882 | 2,211 |
|  |  |  | rating2 | 9 | 10 | 1 | 1 | 3 | 8 | 1,44 | 4,30 | 0,726 | 2,627 |
|  |  | V3 | rating1 | 8 | 12 | 1 | 3 | 3 | 9 | 1,38 | 6,42 | 0,744 | 1,881 |
|  |  |  | rating2 | 8 | 12 | 1 | 3 | 6 | 8 | 1,88 | 6,17 | 1,808 | 1,850 |
| 17 | Spider Videos | V1 | rating1 | 10 | 13 | 1 | 3 | 4 | 9 | 1,60 | 6,54 | 0,966 | 1,761 |
|  |  |  | rating2 | 10 | 13 | 1 | 1 | 3 | 8 | 1,30 | 6,00 | 0,675 | 2,160 |
|  |  | V2 | rating1 | 9 | 10 | 1 | 2 | 2 | 8 | 1,33 | 5,50 | 0,500 | 1,958 |
|  |  |  | rating2 | 9 | 10 | 1 | 1 | 3 | 8 | 1,33 | 4,60 | 0,707 | 2,757 |
|  |  | V3 | rating1 | 8 | 12 | 1 | 3 | 3 | 8 | 1,50 | 6,17 | 0,756 | 1,528 |
|  |  |  | rating2 | 8 | 12 | 1 | 2 | 5 | 8 | 1,63 | 5,33 | 1,408 | 1,775 |
| 18 | Spider Videos | V1 | rating1 | 10 | 13 | 1 | 2 | 4 | 8 | 1,70 | 5,00 | 1,160 | 2,082 |
|  |  |  | rating2 | 10 | 13 | 1 | 1 | 4 | 8 | 1,40 | 4,46 | 0,966 | 2,295 |
|  |  | V2 | rating1 | 9 | 10 | 1 | 2 | 3 | 8 | 1,56 | 5,20 | 0,726 | 2,150 |
|  |  |  | rating2 | 9 | 10 | 1 | 1 | 3 | 9 | 1,67 | 4,40 | 1,000 | 2,716 |
|  |  | V3 | rating1 | 8 | 12 | 1 | 2 | 3 | 9 | 1,38 | 5,83 | 0,744 | 2,082 |
|  |  |  | rating2 | 8 | 12 | 1 | 2 | 6 | 9 | 1,75 | 5,33 | 1,753 | 2,060 |

| Video | | | | N | | min | | max | | mean | | SD | |
| --- | --- | --- | --- | --- | --- | --- | --- | --- | --- | --- | --- | --- | --- |
|  |  |  |  | HC | PP | HC | PP | HC | PP | HC | PP | HC | PP |
| 19 | Spider Videos | V1 | rating1 | 10 | 13 | 1 | 1 | 4 | 8 | 1,80 | 5,92 | 1,135 | 2,178 |
|  |  |  | rating2 | 10 | 13 | 1 | 1 | 2 | 9 | 1,10 | 5,77 | 0,316 | 2,242 |
|  |  | V2 | rating1 | 9 | 10 | 1 | 2 | 3 | 8 | 1,22 | 5,40 | 0,667 | 2,319 |
|  |  |  | rating2 | 9 | 10 | 1 | 1 | 5 | 8 | 1,56 | 4,70 | 1,333 | 2,541 |
|  |  | V3 | rating1 | 8 | 12 | 1 | 4 | 3 | 9 | 1,50 | 7,00 | 0,926 | 1,706 |
|  |  |  | rating2 | 8 | 12 | 1 | 4 | 8 | 9 | 2,13 | 6,50 | 2,475 | 1,834 |
| 20 | Spider Videos | V1 | rating1 | 10 | 13 | 1 | 3 | 3 | 9 | 1,50 | 6,31 | 0,850 | 1,750 |
|  |  |  | rating2 | 10 | 13 | 1 | 1 | 2 | 8 | 1,10 | 5,85 | 0,316 | 2,193 |
|  |  | V2 | rating1 | 9 | 10 | 1 | 2 | 3 | 8 | 1,44 | 5,10 | 0,726 | 2,234 |
|  |  |  | rating2 | 9 | 10 | 1 | 1 | 5 | 9 | 1,78 | 4,10 | 1,394 | 2,470 |
|  |  | V3 | rating1 | 8 | 12 | 1 | 3 | 4 | 8 | 1,50 | 5,33 | 1,069 | 1,557 |
|  |  |  | rating2 | 8 | 12 | 1 | 2 | 8 | 7 | 2,00 | 4,50 | 2,449 | 1,834 |
| 21 | Control Videos | V1 | rating1 | 10 | 13 | 1 | 1 | 2 | 2 | 1,10 | 1,23 | 0,316 | 0,439 |
|  |  |  | rating2 | 10 | 13 | 1 | 1 | 3 | 2 | 1,20 | 1,08 | 0,632 | 0,277 |
|  |  | V2 | rating1 | 9 | 10 | 1 | 1 | 1 | 3 | 1,00 | 1,20 | 0,000 | 0,632 |
|  |  |  | rating2 | 9 | 10 | 1 | 1 | 1 | 1 | 1,00 | 1,00 | 0,000 | 0,000 |
|  |  | V3 | rating1 | 8 | 12 | 1 | 1 | 2 | 2 | 1,13 | 1,17 | 0,354 | 0,389 |
|  |  |  | rating2 | 8 | 12 | 1 | 1 | 2 | 3 | 1,13 | 1,17 | 0,354 | 0,577 |
| 22 | Control Videos | V1 | rating1 | 10 | 13 | 1 | 1 | 3 | 2 | 1,20 | 1,15 | 0,632 | 0,376 |
|  |  |  | rating2 | 10 | 13 | 1 | 1 | 2 | 3 | 1,10 | 1,15 | 0,316 | 0,555 |
|  |  | V2 | rating1 | 9 | 10 | 1 | 1 | 1 | 2 | 1,00 | 1,10 | 0,000 | 0,316 |
|  |  |  | rating2 | 9 | 10 | 1 | 1 | 1 | 1 | 1,00 | 1,00 | 0,000 | 0,000 |
|  |  | V3 | rating1 | 8 | 12 | 1 | 1 | 1 | 1 | 1,00 | 1,00 | 0,000 | 0,000 |
|  |  |  | rating2 | 8 | 12 | 1 | 1 | 1 | 1 | 1,00 | 1,00 | 0,000 | 0,000 |
| 23 | Control Videos | V1 | rating1 | 10 | 13 | 1 | 1 | 5 | 2 | 1,60 | 1,08 | 1,265 | 0,277 |
|  |  |  | rating2 | 10 | 13 | 1 | 1 | 4 | 1 | 1,30 | 1,00 | 0,949 | 0,000 |
|  |  | V2 | rating1 | 9 | 10 | 1 | 1 | 1 | 4 | 1,00 | 1,40 | 0,000 | 0,966 |
|  |  |  | rating2 | 9 | 10 | 1 | 1 | 1 | 2 | 1,00 | 1,10 | 0,000 | 0,316 |
|  |  | V3 | rating1 | 8 | 12 | 1 | 1 | 1 | 1 | 1,00 | 1,00 | 0,000 | 0,000 |
|  |  |  | rating2 | 8 | 12 | 1 | 1 | 1 | 1 | 1,00 | 1,00 | 0,000 | 0,000 |
| 24 | Control Videos | V1 | rating1 | 10 | 13 | 1 | 1 | 4 | 4 | 1,50 | 1,31 | 0,972 | 0,855 |
|  |  |  | rating2 | 10 | 13 | 1 | 1 | 5 | 4 | 1,40 | 1,23 | 1,265 | 0,832 |
|  |  | V2 | rating1 | 9 | 10 | 1 | 1 | 1 | 1 | 1,00 | 1,00 | 0,000 | 0,000 |
|  |  |  | rating2 | 9 | 10 | 1 | 1 | 1 | 1 | 1,00 | 1,00 | 0,000 | 0,000 |
|  |  | V3 | rating1 | 8 | 12 | 1 | 1 | 1 | 1 | 1,00 | 1,00 | 0,000 | 0,000 |
|  |  |  | rating2 | 8 | 12 | 1 | 1 | 1 | 1 | 1,00 | 1,00 | 0,000 | 0,000 |

| Video | | | | N | | min | | max | | mean | | SD | |
| --- | --- | --- | --- | --- | --- | --- | --- | --- | --- | --- | --- | --- | --- |
|  |  |  |  | HC | PP | HC | PP | HC | PP | HC | PP | HC | PP |
| 25 | Control Videos | V1 | rating1 | 10 | 13 | 1 | 1 | 3 | 2 | 1,30 | 1,08 | 0,675 | 0,277 |
|  |  |  | rating2 | 10 | 13 | 1 | 1 | 1 | 1 | 1,00 | 1,00 | 0,000 | 0,000 |
|  |  | V2 | rating1 | 9 | 10 | 1 | 1 | 1 | 1 | 1,00 | 1,00 | 0,000 | 0,000 |
|  |  |  | rating2 | 9 | 10 | 1 | 1 | 1 | 1 | 1,00 | 1,00 | 0,000 | 0,000 |
|  |  | V3 | rating1 | 8 | 12 | 1 | 1 | 1 | 1 | 1,00 | 1,00 | 0,000 | 0,000 |
|  |  |  | rating2 | 8 | 12 | 1 | 1 | 1 | 1 | 1,00 | 1,00 | 0,000 | 0,000 |
| 26 | Control Videos | V1 | rating1 | 10 | 13 | 1 | 1 | 3 | 2 | 1,20 | 1,08 | 0,632 | 0,277 |
|  |  |  | rating2 | 10 | 13 | 1 | 1 | 2 | 1 | 1,10 | 1,00 | 0,316 | 0,000 |
|  |  | V2 | rating1 | 9 | 10 | 1 | 1 | 1 | 1 | 1,00 | 1,00 | 0,000 | 0,000 |
|  |  |  | rating2 | 9 | 10 | 1 | 1 | 1 | 1 | 1,00 | 1,00 | 0,000 | 0,000 |
|  |  | V3 | rating1 | 8 | 12 | 1 | 1 | 1 | 1 | 1,00 | 1,00 | 0,000 | 0,000 |
|  |  |  | rating2 | 8 | 12 | 1 | 1 | 1 | 1 | 1,00 | 1,00 | 0,000 | 0,000 |
| 27 | Control Videos | V1 | rating1 | 10 | 13 | 1 | 1 | 3 | 3 | 1,40 | 1,15 | 0,843 | 0,555 |
|  |  |  | rating2 | 10 | 13 | 1 | 1 | 4 | 2 | 1,30 | 1,08 | 0,949 | 0,277 |
|  |  | V2 | rating1 | 9 | 10 | 1 | 1 | 3 | 3 | 1,22 | 1,40 | 0,667 | 0,699 |
|  |  |  | rating2 | 9 | 10 | 1 | 1 | 4 | 2 | 1,44 | 1,10 | 1,014 | 0,316 |
|  |  | V3 | rating1 | 8 | 12 | 1 | 1 | 1 | 1 | 1,00 | 1,00 | 0,000 | 0,000 |
|  |  |  | rating2 | 8 | 12 | 1 | 1 | 1 | 1 | 1,00 | 1,00 | 0,000 | 0,000 |
| 28 | Control Videos | V1 | rating1 | 10 | 13 | 1 | 1 | 2 | 1 | 1,30 | 1,00 | 0,483 | 0,000 |
|  |  |  | rating2 | 10 | 13 | 1 | 1 | 3 | 2 | 1,20 | 1,15 | 0,632 | 0,376 |
|  |  | V2 | rating1 | 9 | 10 | 1 | 1 | 1 | 1 | 1,00 | 1,00 | 0,000 | 0,000 |
|  |  |  | rating2 | 9 | 10 | 1 | 1 | 1 | 1 | 1,00 | 1,00 | 0,000 | 0,000 |
|  |  | V3 | rating1 | 8 | 12 | 1 | 1 | 1 | 1 | 1,00 | 1,00 | 0,000 | 0,000 |
|  |  |  | rating2 | 8 | 12 | 1 | 1 | 1 | 1 | 1,00 | 1,00 | 0,000 | 0,000 |
| 29 | Control Videos | V1 | rating1 | 10 | 13 | 1 | 1 | 3 | 2 | 1,30 | 1,08 | 0,675 | 0,277 |
|  |  |  | rating2 | 10 | 13 | 1 | 1 | 4 | 2 | 1,30 | 1,08 | 0,949 | 0,277 |
|  |  | V2 | rating1 | 9 | 10 | 1 | 1 | 1 | 1 | 1,00 | 1,00 | 0,000 | 0,000 |
|  |  |  | rating2 | 9 | 10 | 1 | 1 | 1 | 1 | 1,00 | 1,00 | 0,000 | 0,000 |
|  |  | V3 | rating1 | 8 | 12 | 1 | 1 | 1 | 1 | 1,00 | 1,00 | 0,000 | 0,000 |
|  |  |  | rating2 | 8 | 12 | 1 | 1 | 1 | 1 | 1,00 | 1,00 | 0,000 | 0,000 |
| 30 | Control Videos | V1 | rating1 | 10 | 13 | 1 | 1 | 3 | 1 | 1,40 | 1,00 | 0,699 | 0,000 |
|  |  |  | rating2 | 10 | 13 | 1 | 1 | 2 | 1 | 1,10 | 1,00 | 0,316 | 0,000 |
|  |  | V2 | rating1 | 9 | 10 | 1 | 1 | 1 | 2 | 1,00 | 1,10 | 0,000 | 0,316 |
|  |  |  | rating2 | 9 | 10 | 1 | 1 | 1 | 1 | 1,00 | 1,00 | 0,000 | 0,000 |
|  |  | V3 | rating1 | 8 | 12 | 1 | 1 | 1 | 1 | 1,00 | 1,00 | 0,000 | 0,000 |
|  |  |  | rating2 | 8 | 12 | 1 | 1 | 1 | 1 | 1,00 | 1,00 | 0,000 | 0,000 |

| Video | | | | N | | min | | max | | mean | | SD | |
| --- | --- | --- | --- | --- | --- | --- | --- | --- | --- | --- | --- | --- | --- |
|  |  |  |  | HC | PP | HC | PP | HC | PP | HC | PP | HC | PP |
| 31 | Control Videos | V1 | rating1 | 10 | 13 | 1 | 1 | 1 | 1 | 1,00 | 1,00 | 0,000 | 0,000 |
|  |  |  | rating2 | 10 | 13 | 1 | 1 | 2 | 1 | 1,10 | 1,00 | 0,316 | 0,000 |
|  |  | V2 | rating1 | 9 | 10 | 1 | 1 | 1 | 2 | 1,00 | 1,10 | 0,000 | 0,316 |
|  |  |  | rating2 | 9 | 10 | 1 | 1 | 1 | 1 | 1,00 | 1,00 | 0,000 | 0,000 |
|  |  | V3 | rating1 | 8 | 12 | 1 | 1 | 1 | 1 | 1,00 | 1,00 | 0,000 | 0,000 |
|  |  |  | rating2 | 8 | 12 | 1 | 1 | 1 | 1 | 1,00 | 1,00 | 0,000 | 0,000 |
| 32 | Control Videos | V1 | rating1 | 10 | 13 | 1 | 1 | 3 | 2 | 1,30 | 1,15 | 0,675 | 0,376 |
|  |  |  | rating2 | 10 | 13 | 1 | 1 | 2 | 1 | 1,10 | 1,00 | 0,316 | 0,000 |
|  |  | V2 | rating1 | 9 | 10 | 1 | 1 | 1 | 2 | 1,00 | 1,20 | 0,000 | 0,422 |
|  |  |  | rating2 | 9 | 10 | 1 | 1 | 1 | 1 | 1,00 | 1,00 | 0,000 | 0,000 |
|  |  | V3 | rating1 | 8 | 12 | 1 | 1 | 1 | 1 | 1,00 | 1,00 | 0,000 | 0,000 |
|  |  |  | rating2 | 8 | 12 | 1 | 1 | 1 | 1 | 1,00 | 1,00 | 0,000 | 0,000 |
| 33 | Control Videos | V1 | rating1 | 10 | 13 | 1 | 1 | 2 | 2 | 1,10 | 1,08 | 0,316 | 0,277 |
|  |  |  | rating2 | 10 | 13 | 1 | 1 | 1 | 1 | 1,00 | 1,00 | 0,000 | 0,000 |
|  |  | V2 | rating1 | 9 | 10 | 1 | 1 | 1 | 2 | 1,00 | 1,10 | 0,000 | 0,316 |
|  |  |  | rating2 | 9 | 10 | 1 | 1 | 1 | 2 | 1,00 | 1,10 | 0,000 | 0,316 |
|  |  | V3 | rating1 | 8 | 12 | 1 | 1 | 1 | 1 | 1,00 | 1,00 | 0,000 | 0,000 |
|  |  |  | rating2 | 8 | 12 | 1 | 1 | 1 | 2 | 1,00 | 1,08 | 0,000 | 0,289 |
| 34 | Control Videos | V1 | rating1 | 10 | 13 | 1 | 1 | 3 | 3 | 1,20 | 1,15 | 0,632 | 0,555 |
|  |  |  | rating2 | 10 | 13 | 1 | 1 | 3 | 2 | 1,20 | 1,08 | 0,632 | 0,277 |
|  |  | V2 | rating1 | 9 | 10 | 1 | 1 | 1 | 3 | 1,00 | 1,20 | 0,000 | 0,632 |
|  |  |  | rating2 | 9 | 10 | 1 | 1 | 1 | 1 | 1,00 | 1,00 | 0,000 | 0,000 |
|  |  | V3 | rating1 | 8 | 12 | 1 | 1 | 1 | 1 | 1,00 | 1,00 | 0,000 | 0,000 |
|  |  |  | rating2 | 8 | 12 | 1 | 1 | 1 | 1 | 1,00 | 1,00 | 0,000 | 0,000 |
| 35 | Control Videos | V1 | rating1 | 10 | 13 | 1 | 1 | 1 | 2 | 1,00 | 1,08 | 0,000 | 0,277 |
|  |  |  | rating2 | 10 | 13 | 1 | 1 | 2 | 1 | 1,10 | 1,00 | 0,316 | 0,000 |
|  |  | V2 | rating1 | 9 | 10 | 1 | 1 | 1 | 2 | 1,00 | 1,20 | 0,000 | 0,422 |
|  |  |  | rating2 | 9 | 10 | 1 | 1 | 2 | 2 | 1,11 | 1,10 | 0,333 | 0,316 |
|  |  | V3 | rating1 | 8 | 12 | 1 | 1 | 1 | 2 | 1,00 | 1,08 | 0,000 | 0,289 |
|  |  |  | rating2 | 8 | 12 | 1 | 1 | 1 | 2 | 1,00 | 1,08 | 0,000 | 0,289 |
| 36 | Control Videos | V1 | rating1 | 10 | 13 | 1 | 1 | 2 | 2 | 1,10 | 1,08 | 0,316 | 0,277 |
|  |  |  | rating2 | 10 | 13 | 1 | 1 | 1 | 1 | 1,00 | 1,00 | 0,000 | 0,000 |
|  |  | V2 | rating1 | 9 | 10 | 1 | 1 | 2 | 4 | 1,11 | 1,30 | 0,333 | 0,949 |
|  |  |  | rating2 | 9 | 10 | 1 | 1 | 2 | 4 | 1,11 | 1,30 | 0,333 | 0,949 |
|  |  | V3 | rating1 | 8 | 12 | 1 | 1 | 1 | 2 | 1,00 | 1,08 | 0,000 | 0,289 |
|  |  |  | rating2 | 8 | 12 | 1 | 1 | 2 | 2 | 1,13 | 1,08 | 0,354 | 0,289 |

| Video | | | | N | | min | | max | | mean | | SD | |
| --- | --- | --- | --- | --- | --- | --- | --- | --- | --- | --- | --- | --- | --- |
|  |  |  |  | HC | PP | HC | PP | HC | PP | HC | PP | HC | PP |
| 37 | Control Videos | V1 | rating1 | 10 | 13 | 1 | 1 | 3 | 1 | 1,20 | 1,00 | 0,632 | 0,000 |
|  |  |  | rating2 | 10 | 13 | 1 | 1 | 4 | 2 | 1,30 | 1,08 | 0,949 | 0,277 |
|  |  | V2 | rating1 | 9 | 10 | 1 | 1 | 1 | 1 | 1,00 | 1,00 | 0,000 | 0,000 |
|  |  |  | rating2 | 9 | 10 | 1 | 1 | 1 | 1 | 1,00 | 1,00 | 0,000 | 0,000 |
|  |  | V3 | rating1 | 8 | 12 | 1 | 1 | 1 | 3 | 1,00 | 1,17 | 0,000 | 0,577 |
|  |  |  | rating2 | 8 | 12 | 1 | 1 | 1 | 2 | 1,00 | 1,08 | 0,000 | 0,289 |
| 38 | Control Videos | V1 | rating1 | 10 | 13 | 1 | 1 | 2 | 2 | 1,30 | 1,08 | 0,483 | 0,277 |
|  |  |  | rating2 | 10 | 13 | 1 | 1 | 3 | 2 | 1,20 | 1,08 | 0,632 | 0,277 |
|  |  | V2 | rating1 | 9 | 10 | 1 | 1 | 1 | 1 | 1,00 | 1,00 | 0,000 | 0,000 |
|  |  |  | rating2 | 9 | 10 | 1 | 1 | 1 | 1 | 1,00 | 1,00 | 0,000 | 0,000 |
|  |  | V3 | rating1 | 8 | 12 | 1 | 1 | 2 | 1 | 1,25 | 1,00 | 0,463 | 0,000 |
|  |  |  | rating2 | 8 | 12 | 1 | 1 | 1 | 1 | 1,00 | 1,00 | 0,000 | 0,000 |
| 39 | Control Videos | V1 | rating1 | 10 | 13 | 1 | 1 | 2 | 3 | 1,20 | 1,23 | 0,422 | 0,599 |
|  |  |  | rating2 | 10 | 13 | 1 | 1 | 1 | 2 | 1,00 | 1,15 | 0,000 | 0,376 |
|  |  | V2 | rating1 | 9 | 10 | 1 | 1 | 1 | 3 | 1,00 | 1,20 | 0,000 | 0,632 |
|  |  |  | rating2 | 9 | 10 | 1 | 1 | 1 | 2 | 1,00 | 1,10 | 0,000 | 0,316 |
|  |  | V3 | rating1 | 8 | 12 | 1 | 1 | 1 | 1 | 1,00 | 1,00 | 0,000 | 0,000 |
|  |  |  | rating2 | 8 | 12 | 1 | 1 | 1 | 1 | 1,00 | 1,00 | 0,000 | 0,000 |
| 40 | Control Videos | V1 | rating1 | 10 | 13 | 1 | 1 | 3 | 1 | 1,30 | 1,00 | 0,675 | 0,000 |
|  |  |  | rating2 | 10 | 13 | 1 | 1 | 2 | 2 | 1,10 | 1,08 | 0,316 | 0,277 |
|  |  | V2 | rating1 | 9 | 10 | 1 | 1 | 1 | 3 | 1,00 | 1,30 | 0,000 | 0,675 |
|  |  |  | rating2 | 9 | 10 | 1 | 1 | 1 | 2 | 1,00 | 1,10 | 0,000 | 0,316 |
|  |  | V3 | rating1 | 8 | 12 | 1 | 1 | 2 | 2 | 1,13 | 1,17 | 0,354 | 0,389 |
|  |  |  | rating2 | 8 | 12 | 1 | 1 | 1 | 2 | 1,00 | 1,08 | 0,000 | 0,289 |

**Supplementary Figure S1:** Placement of optodes and electrodes on the cap. Red circles mark NIRS emitters, blue detectors. Note that the combined fNIRS-EEG cap is able to measure more electrode positions (e.g. Fp1, Fpz, Fp2) as were realized in this study.


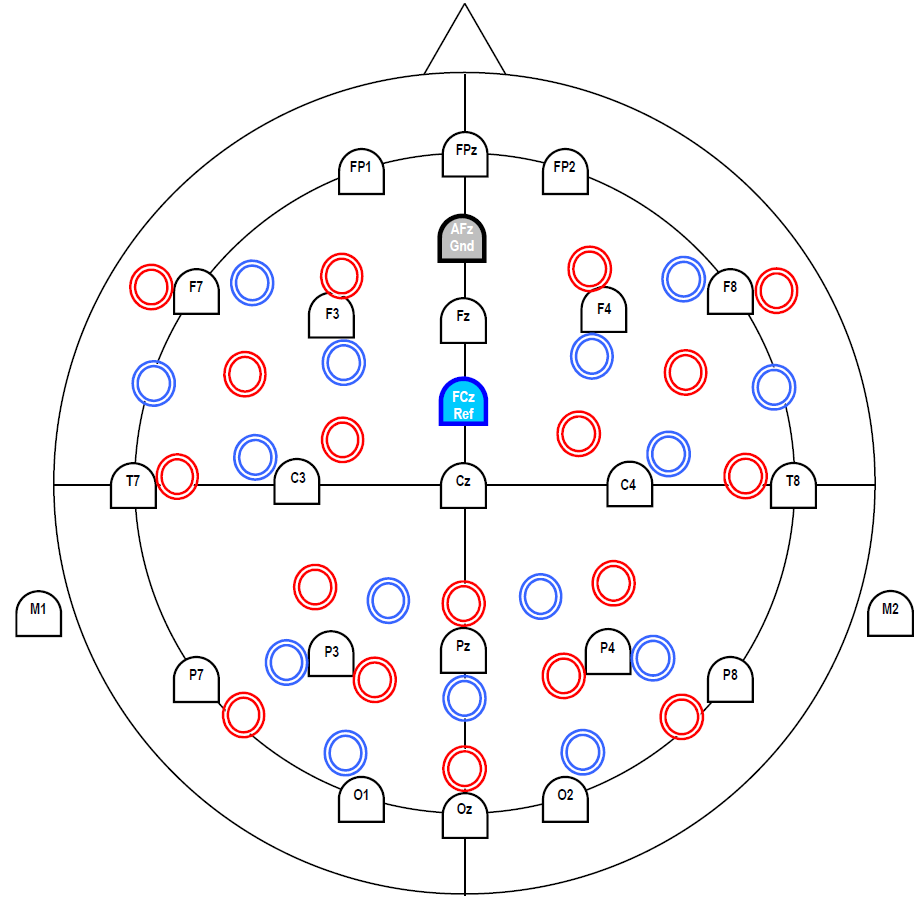

Supplement: Supplementary file 1 — Supplementary file1 [file 41598_2020_69127_MOESM1_ESM.docx]
